# Chemotherapy-elicited exosomal miR-378a-3p and miR-378d promote breast cancer stemness and chemoresistance via the activation of EZH2/STAT3 signaling

**DOI:** 10.1186/s13046-021-01901-1

**Published:** 2021-04-06

**Authors:** Qianxi Yang, Shaorong Zhao, Zhendong Shi, Lixia Cao, Jingjing Liu, Teng Pan, Dongdong Zhou, Jin Zhang

**Affiliations:** 1 Third Department of Breast Surgery, Tianjin Medical University Cancer Institute and Hospital, Tianjin, China; 2 National Clinical Research Center for Cancer, Tianjin, China; 3 Key Laboratory of Cancer Prevention and Therapy, Tianjin, China; 4 Clinical Research Center for Cancer, Tianjin, China

**Keywords:** Chemotherapy-elicited exosomes, miR-378a-3p, miR-378d, Chemotherapy resistance, Cancer stemness

## Abstract

**Background:**

Not all breast cancer (BC) patients who receive neoadjuvant chemotherapy achieve a pathologic complete response (pCR), but the reasons for this are unknown. Previous studies have shown that exosomes produced in the tumor microenvironment in response to chemotherapy promote a chemotherapy-resistant phenotype in tumors. However, the role of BC chemotherapy-elicited exosomes in regulating chemoresistance is poorly understood.

**Methods:**

Using commercial kits, serum exosomes were extracted from patients before neoadjuvant chemotherapy, after one cycle of chemotherapy and after four cycles of chemotherapy consisting of doxorubicin (DOX) and paclitaxel (PTX). Their miRNAs were sequenced, and the correlation between the sequencing results and chemotherapy effects was further verified by RT-qPCR using patient serum exosomes. Cell Counting Kit-8 (CCK-8) was used to detect chemosensitivity. Stemness was assessed by CD44+/CD24- population analysis and mammosphere formation assays. Chromatin immunoprecipitation (ChIP) experiments were performed to verify the binding of signal transducer and activator of transcription 3 (STAT3) to the promoter of miRNAs.

**Results:**

Here, we provide clinical evidence that chemotherapy-elicited exosomal miR-378a-3p and miR-378d are closely related to the chemotherapy response and that exosomes produced by BC cells after stimulation with DOX or PTX deliver miR-378a-3p and miR-378d to neighboring cells to activate WNT and NOTCH stemness pathways and induce drug resistance by targeting Dickkopf 3 (DKK3) and NUMB. In addition, STAT3, which is enhanced by zeste homolog 2 (EZH2), bound to the promoter regions of miR-378a-3p and miR-378d, thereby increasing their expression in exosomes. More importantly, chemotherapeutic agents combined with the EZH2 inhibitor tazemetostat reversed chemotherapy-elicited exosome-induced drug resistance in a nude mouse tumor xenograft model.

**Conclusion:**

This study revealed a novel mechanism of acquired chemoresistance whereby chemotherapy activates the EZH2/STAT3 axis in BC cells, which then secrete chemotherapy-elicited exosomes enriched in miR-378a-3p and miR-378d. These exosomes are absorbed by chemotherapy-surviving BC cells, leading to activation of the WNT and NOTCH stem cell pathways via the targeting of DKK3 and NUMB and subsequently resulting in drug resistance. Therefore, blocking this adaptive mechanism during chemotherapy may reduce the development of chemotherapy resistance and maximize the therapeutic effect.

**Supplementary Information:**

The online version contains supplementary material available at 10.1186/s13046-021-01901-1.

## Background

Neoadjuvant chemotherapy is the most common method of systemic treatment administered to shrink tumors and destroy undetected metastatic cells, thereby facilitating subsequent surgery or radiation therapy [1]. However, a disadvantage of this therapy is that it may not kill all of the tumor cells, which leads to increased resistance of the remaining cells to subsequent chemotherapy [2].

Exosomes, which have recently become a research focus in tumor biology, are extracellular vesicles (diameter, 30–150 nm) that contain lipids, proteins and nucleic acids and play a role in intercellular communication [3]. Many recent studies have shown that drug-resistant cancer cells in tumors release exosomal miRNAs that directly induce the conversion of sensitive cells to their drug-resistant counterparts or deliver miRNAs that interact with cells in the tumor microenvironment to modulate the drug-resistance response in that region [4]. As a novel mechanism of drug resistance, chemotherapy has been shown to stimulate tumor cells to secrete exosomes enriched in ANXA6, which deliver heterogeneous and enhance the capacity for transfer [5] and promote cancer stemness by targeting ONECUT2 [6]. However, the specific mechanism of action and clinical relevance of chemotherapy-elicited exosomes have yet to be investigated.

Recent studies have identified the crucial role of stem-like tumor-initiating cell (TIC) expansion in mediating tumor therapeutic resistance and metastasis [7]. Enhancer of zeste homolog 2 (EZH2) promotes the chemoresistance of breast cancer [8] and other types of cancer [9] by inducing the expansion of TICs. EZH2 also acts as a stemness factor and drives oncogenic dedifferentiation [10]. In addition, STAT3 plays a critical role in breast cancer chemoresistance and stem cell self-renewal. For example, the JAK/STAT3-mediated regulation of lipid metabolism is essential for breast cancer stem cell (BCSC) self-renewal and cancer chemoresistance [11]. EZH2 binds to STAT3, which leads to enhanced STAT3 activity via the increased tyrosine phosphorylation of STAT3 in cancer stem cells (CSCs) [12].

In this study, we demonstrate that two classes of cytotoxic agents widely used in neoadjuvant breast cancer therapy, including taxanes and anthracyclines, can stimulate cancer cells to secrete exosomes that suppress chemosensitivity by promoting the expansion of TICs. Increased EZH2 in cancer cells after chemotherapy activated the transcription factor STAT3 and bound to the promoter regions of miR-378a-3p and miR-378d. This induced the secretion of exosomes enriched in miR-378a-3p and miR-378d and subsequent modulation of multiple signaling pathways, including WNT/β-catenin and Notch, through the direct targeting of the WNT antagonist DKK3 and the Notch suppressor NUMB. As a result, there was a substantial increase in the number of breast cancer TICs. These studies indicate that chemotherapy in combination with EZH2 inhibitors is a highly promising method for improving drug efficacy and reversing drug resistance. In addition, these findings highlight the need for more investigations on chemotherapy-elicited exosome-mediated drug resistance.

## Methods

### Cell culture and transfection

The human breast cancer cell lines CAL51, MDA-MB-231 and MCF-7 were obtained from the Chinese Science Institute. The CAL51, MDA-MB-231 cells were cultured in RPMI 1640 (RPMI 1640; CORNING), and the MCF-7 cells were cultured in DMEM (DMEM; CORNING) and all supplemented with 10% fetal bovine serum. Cells were cultivated at 37 °C in a humidified chamber supplemented with 5% CO2.

MiRNA mimics and inhibitors and their negative control counterparts were obtained from GenePharma, China, and their sequences are shown in Table S1. DKK3 and NUMB overexpression plasmids and a negative control plasmid were purchased from Yi Xiang, China. EZH2 and STAT3 siRNA and negative control siRNA were purchased from Ribobio, China. Transient transfection with RNA and DNA was performed using Lipofectamine 3000 (Thermo Fisher, USA) in accordance with the manufacturer’s protocols. Cells (2 × 10^5^) were seeded into 6-well plates one day before transfection with 25 pmol siRNA/miRNA mimics/miRNA inhibitors or 2 mg plasmid DNA using 7.5 μL Lipofectamine.

### Exosome purification and validation

Serum exosomes were isolated using an exosome isolation kit (Thermo Fisher Scientific, USA). Exosomes secreted into the cell culture supernatant were purified. First, conditioned medium was prepared from CAL51, MDA-MB-231 or MCF7 cells cultured for 4 h in medium containing doxorubicin (DOX 75 nmol/L) (Sigma-Aldrich, USA), paclitaxel (PTX 100 nmol/L) (Sigma-Aldrich, USA) or PBS as the control. The cells were washed with PBS and then added to growth medium supplemented with fetal bovine serum (FBS) (ultracentrifugation at 100,000×*g* overnight at 4 °C) without exosomes for 48 h. The culture supernatant was centrifuged at 2000×*g* for 10 min and then 10,000×*g* for 20 min to remove cell debris and vesicles. Exosomes were extracted by ultracentrifugation at 110,000×*g* for 70 min and washed with PBS under the same ultracentrifugation conditions. The purified exosomes were resuspended in PBS and characterized by electron microscopy and western blotting. The BCA Protein Assay Kit (Thermo Fisher Scientific, USA) was used to quantify exosome proteins, and exosomes collected from 5 × 10^6^ producer cells (approximately 0.4 mg of exosomes) were added to 5 × 10^5^ recipient cells. To fluorescently label exosomes, resuspended exosomes were incubated with 20 mmol/L PKH26 (Sigma-Aldrich, USA) at 37 °C for 2 h before washing in PBS.

### Tumor xenograft

All mice used in this study were housed in pathogen-free barrier animal facilities that met the national regulations for the use and care of mice in experimental studies. Research involving mice complied with all relevant ethical regulations governing animal research. The experiments were performed at the Tianjin Medical University Cancer Institute and Hospital Laboratory. All animal experiments were approved by the Tianjin Medical University Cancer Institute and Hospital Laboratory Animal Care and Use Committee.

In vivo experiments to study chemotherapy-induced drug resistance in exosomes were conducted in a nude mouse tumor xenograft model established by injecting 2 × 10^6^ CAL51 cells into the mammary fat pad (#4) of 20 mice. When the tumor size reached approximately 50 mm^3^, the mice were randomly divided into two equal groups. Chemo-naïve CAL51 exosomes or DOX chemotherapy-elicited CAL51 exosomes (20 μg) were then injected around the tumors in mice every 5 days for a total of 15 days. When the tumor size reached 80–100 mm^3^, mice that received the same treatments were randomly divided into two equal groups and administered a tail vein injection of DOX (4 mg/kg) or PBS every 5 days for 3 weeks.

In vivo studies were conducted to investigate the combined effects of tazemetostat (Selleck, USA) and doxorubicin (DOX) (Sigma-Aldrich, USA). Xenografted tumors were established in NSG mice by injecting 2 × 10^6^ CAL51 cells into the mammary fat pad (#4) of 20 mice. The tumor volume was determined using Vernier calipers, and when the tumor size reached approximately 40 mm^3^, DOX chemotherapy-elicited CAL51 exosomes (20 μg) were injected around the tumors in mice every 5 days for a total of 15 days. When the tumor size reached 80–100 mm^3^, the mice were randomly divided into four equal groups and treated with PBS, tazemetostat (200 mg/kg), DOX (4 mg/kg) or tazemetostat (200 mg/kg) + DOX (4 mg/kg) every 5 days for 3 weeks. Tumors were collected 3 days after the last treatment. DOX was injected into the tail vein, and tazemetostat was administered orally.

In vivo studies were conducted to verify the effects of EZH2 overexpression on exosome-miRNAs and chemotherapy. We constructed a CAL51-EZH2 stable overexpression cell line and control CAL51-MCS cell line. The mammary fat pad (#4) of mice (*n* = 20) were injected with 2 × 10^6^ CAL51-EZH2 cells, and the mice were randomly divided into two equal groups. Chemo-naïve CAL51 exosomes or DOX chemotherapy-elicited CAL51 exosomes (20 μg) were injected around the tumors every 5 days for a total of 15 days. When the tumor size reached 80–100 mm^3^, the mice that received the same treatments were randomly divided into two equal groups and administered a tail vein injection of DOX (4 mg/kg) or PBS every 5 days for 3 weeks. Another group of  mice (*n* = 20) received an injection of 2 × 10^6^ CAL51-MCS cells into their mammary fat pad (#4), and the same experiments as described above were performed after tumor formation.

The tumor size was measured using vernier calipers. And the serum from each experimental mouse model was collected through retro-orbital bleeding after the treatment was complete. Each tumor was cut into two sections for western blot and immunohistochemistry (IHC) analyses.

### Clinical specimens

Human tumor specimens were obtained from breast cancer patients who had received 4–8 neoadjuvant chemotherapy cycles consisting of anthracyclines combined with or followed by paclitaxel in accordance with the clinical protocols at Tianjin Medical University Cancer Hospital. All patients provided written informed consent to participate in this study. The studies were conducted in accordance with accepted ethical guidelines and approved by the Institutional Review Board. The clinical characteristics of patients, including pathology, age, lymph node metastasis, tumor size, neoadjuvant regimens and response to chemotherapy, are summarized in Table S2.

### Immunohistochemistry (IHC)

Tumor tissues were fixed with 4% paraformaldehyde, paraffin-embedded, sectioned and stained with anti-DKK3 antibodies (1:100) (Immunoway, YT1355) and anti-NUMB antibodies (1:100) (Immunoway, YT5320). Slides were scored based on the percentage of tumor cells positive for antigen staining and the intensity of staining to obtain a final score for statistical analysis.

### RNA extraction and quantitative RT-qPCR

RNA extraction with TRIzol (ThermoFisher Scientific) and RT-qPCR were performed as described previously. MiRNA was extracted using a miRNeasy Mini Kit (Qiagen, USA) and miRNeasy Serum/Plasma Kit (Qiagen, USA). The primer sequences used in RT-qPCR are indicated in Table S3. The annealing temperature for all primers was 55 °C. For the detection of intracellular miRNAs, U6 small nuclear RNA was used as an internal control. To standardize exosome-miRNAs, equal amounts of exosomes from cell conditioned medium or serum were extracted, and 20 fmol syngeneic cel-miR-39-3p was added during RNA extraction. The data after subsequent miRNA RT-qPCR analysis (Qiagen, USA) were standardized at the level of this spiked control.

### Western blot analysis

The cleaved proteins were resolved by sodium dodecyl sulfate-polyacrylamide electrophoresis (8–12% gel). Selected proteins were detected using the specific antibodies listed in Table S4, and protein levels were normalized against GAPDH.

### Dual-luciferase reporter assay

PCR amplified fragments containing the 3′-UTR of human *DKK3* and *NUMB* were digested with *Xba*I and *Nhe*I and inserted into the pmirGLO reporter vector (Promega, USA). The verification of all plasmid constructs was performed by sequencing. Luciferase activities were measured as described previously [13].

### Three-dimensional (3D) multicellular tumor sphere and mammosphere formation assays

#### 3D multicellular tumor sphere formation assay

First, 96-well plates were coated with agarose, and then cell suspensions containing Matrigel matrix gel (BD Biosciences, USA) were seeded onto the agarose-coated 96-well plates and incubated for 7 days. The formation of multicellular tumor spheres was observed using an inverted microscope. DOX or PTX was then added to the sphere medium, and cells were incubated for an additional 7 days. Changes in the spheres were observed using an inverted microscope with NIS-Elements F software to measure the sphere diameter.

#### Mammosphere formation assay

Cells (2000 cells/well) were seeded in ultra-low adhesion 6-well plates (Corning, USA) and cultured in serum-free DMEM/F12 containing 20 ng/ml EGF, 20 ng/ml bFGF, 4 mg/ml insulin, 0.5% BSA (Sigma-Aldrich, USA) and 2% B27 (Sigma-Aldrich, USA). The medium was replaced every 3 days, and the number of mammospheres was counted after 15 days.

### CD44/CD24 assay

Allophycocyanin (APC)-conjugated anti-CD44 and phycoerythrin (PE)-conjugated anti-CD24 monoclonal antibodies (BD Biosciences, USA) were used to stain stem cell markers. After digestion, the cells were washed with PBS. Subsequently, 1 × 10^6^ cells were resuspended in 100 μl PBS before adding APC-CD44 and its isotype control APC-IgG or PE-CD24 and its isotype control PE-IgG. The cells were incubated at 4 °C for 40 min, washed in PBS and then analyzed using a flow cytometer. Gating parameters were defined according to the cells labeled with the relevant isotype control (APC-IgG and PE-IgG).

### CCK-8 cell cytotoxicity assay

Cell cytotoxicity was analyzed using the Cell Counting Kit-8 (CCK-8) method (MYBiotech, China). Briefly, MDA231, CAL51 and MCF7 cells were co-incubated with extracellular exosomes obtained from the same breast cancer cell line that was exposed to PBS, DOX, PTX, DOX + GW4869, PTX + GW4869 or transfected with miRNA mimics/siRNA. Subsequently, the cells were plated into 96-well plates and treated with DOX or PTX at different doses for 48 h. After drug treatment, 10 μl CCK-8 solution were added to each well, and the cells were incubated at 37 °C for 4 h. The optical density (OD) was measured at 450 nm (ThermoScientific, USA), and the half-maximal inhibitory concentration (IC50) of the drug was calculated based on the OD value. The assay was performed at least three times. Cell cytotoxicity was calculated according to the following formula: inhibition ratio (%) = (OD (drug) - OD (blank))/(OD (drug control) - OD (blank)) × 100%.

### Chromatin immunoprecipitation (ChIP)

ChIP was performed to assess in vivo DNA-protein interactions at the miR-378a-3p and miR-378d promoters using a Magna ChIP A/G Kit in accordance with the manufacturer’s instructions (Millipore, USA). Briefly, the cells were fixed in 1% formaldehyde for 10 min to induce the cross-linking of protein-DNA complexes. After washing with cold PBS, the cells were lysed with cell Lysis Buffer containing Protease Inhibitor Cocktail II. Cells were centrifuged at 4 °C for 5 min at 800×*g*, and the supernatant was removed. Then, cell pellets were resuspended in Nuclear Lysis Buffer containing Protease Inhibitor Cocktail II. After sonicating on wet ice, cell lysates were centrifuged at 10,000×*g* for 10 min at 4 °C, and the supernatant was collected in fresh microfuge tubes. Dilution Buffer containing Protease Inhibitor Cocktail II (450 μL) was added into each tube containing 50 μL of chromatin. Then, 5 μL (1%) of the supernatant were collected and kept at 4 °C for future use as the “Input”. The immunoprecipitating antibody and 20 μL of fully resuspended protein A/G magnetic beads were incubated (with rotation) for 1 h to overnight at 4 °C with 5 μg of STAT3-specific antibodies, 1 μg of the positive control or 1 μg of the negative control. The protein A/G bead-antibody/chromatin complex was washed by resuspending the beads in 0.5 mL of 4 types of cold buffers for 3–5 min and then eluted in 100 μl ChIP elution buffer containing proteinase K. Next, the complexes were incubated at 62 °C for 2 h with shaking and then 95 °C for 10 min. Then, DNA was purified using Spin Columns, and the resulting DNA was stored at − 20 °C until PCR analysis.

### Semiquantitative PCR

The enrichment of miR-378a-3p and miR-378d promoter sequences in ChIP kit-extracted DNA was measured by standard PCR using different primers to generate three 220-bp miR-378a-3p promoter fragments and four 240-bp miR-378d promoter fragments. PCR reactions were performed in a 25 μl volume containing 1 μl ChIP DNA, 0.5 μM of each primer, 12.5 μl 2X EasyTaq PCR SuperMix (TRAN, China) and 10.5 μl Nuclease-free Water. The PCR conditions were as follows: 1 cycle at 94 °C for 5 min, 35 cycles at 94 °C for 30 s, 56 °C for 30 s and 72 °C for 1 min and a final cycle at 72 °C for 5 min. PCR products (15 μl) were taken from each group and run on 0.5 μg/ml GelStain (TRAN, China) stained agarose gels. A Gel Doc XR+ inspection system (Bio-Rad, USA) was used to visualize the bands, and Image Lab software V3.0 (Bio-Rad, USA) was used to capture images. The PCR primers are summarized in Table S5.

### Sequencing analysis of miRNAs isolated from patient serum exosomes

Illumina sequencing analysis of exosomal miRNAs was performed by Novogene using RNA extracted from equal amounts of serum exosomes collected from patients before receiving neoadjuvant chemotherapy, after receiving one cycle of neoadjuvant chemotherapy and after receiving four cycles of neoadjuvant chemotherapy. All small RNAs comprising 15–52 nucleotides were selected and sequenced using the HiSeq™2500/MiSeq system (Illumina) in accordance with the manufacturer’s protocol. Original counts were standardized using the pruned mean mol/L value method, and the results were analyzed using the Bioconductor package “edgeR” to identify the differentially expressed miRNAs for the three types of exosomes.

### Correlation analysis

We calculated the correlation between the expression level of miR-378 and *β-catenin*, *NOTCH1*, *EZH2*, *DKK3*, *NUMB* and *STAT3* protein-coding genes in 470 breast cancer patients using two-sided Pearson correlation coefficients. Additionally, the Pearson correlation coefficients and *p* values were calculated.

### Statistical analysis

All quantitative data were presented as the mean ± standard deviation (SD) and analyzed using GraphPad Prism 8.02. The means of the two data sets were compared using paired t-tests. One-way ANOVA was used to evaluate multiple independent groups. The nonparametric Wilcoxon test was used for the comparison of breast cancer samples before and after neoadjuvant chemotherapy. *P* < 0.05 was set as the threshold for statistical significance. All western blots represent three independent experiments with similar results, and representative images were selected for display.

## Results

### The expression of miR-378a-3p and miR-378d in serum exosomes is increased in breast cancer patients receiving chemotherapy and is associated with chemoresistance

**Fig. 1:**
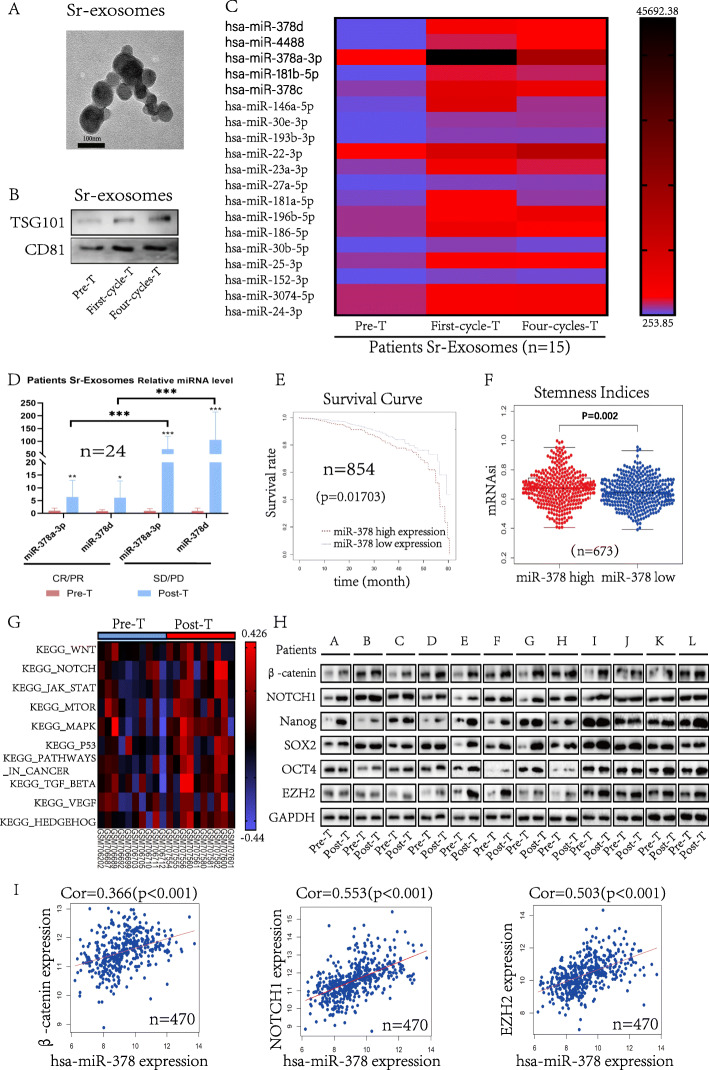
The expression of miR-378a-3p and miR-378d in serum exosomes is increased in breast cancer patients receiving chemotherapy and associated with chemoresistance. **a** Scanning electron microscopy of serum-exosomes isolated from patient serum. **b** Western blot analysis of the key enriched proteins CD81 and TSG101 in breast cancer patient serum exosomes. **c** Sequencing analysis of exosomal microRNAs from patient serum exosomes before receiving neoadjuvant chemotherapy, after receiving one cycle of neoadjuvant chemotherapy and after receiving four cycles of neoadjuvant chemotherapy. **d** Exosomes were isolated from the serum of breast cancer patients before and after neoadjuvant chemotherapy (*n* = 24). Variation in serum exosomal miR-378a-3p and miR-378d in groups with different responses to neoadjuvant chemotherapy. **e** Five-year survival rates in patients with low or high expression miR-378 based on the TCGA database (*n* = 854). **f** Analysis of miR-378 expression and the corresponding novel stemness index mRNAsi in 673 breast cancer patients in the TCGA database. **g** Pre- and post-chemotherapy expression data were analyzed by Gene set variation analysis for the breast cancer cohort (*n* = 10). **h** Western blot analysis of protein expression in breast cancer tissue before and after neoadjuvant chemotherapy in the no response group (*n* = 12). **i** The expression level of miR-378 was positively correlated with *β-catenin*, *NOTCH1* or *EZH2* in breast cancer (*n* = 470). *p < 0.05, ** *p* < 0.01, *** *p* < 0.001

An exosome isolation kit was used to isolate patient serum exosomes. Transmission electron microscopy (Fig. 1a) and analysis of the exosome-associated proteins CD81 and TSG101 (Fig. 1b) demonstrated that highly pure populations of exosomes were obtained. To identify the key exosomal miRNAs related to chemotherapy resistance, we isolated exosomes from 15 patients before receiving neoadjuvant chemotherapy, after receiving one cycle of neoadjuvant chemotherapy and after receiving four cycles of neoadjuvant chemotherapy and then sequenced the miRNAs within these exosomes. The results of differential analyses showed that miR-378a-3p, miR-378c, miR-378d, miR-4488 and miR-181b were substantially increased in serum exosomes after one cycle or four cycles of neoadjuvant chemotherapy (*p* < 0.05) (Fig. 1c and Fig. S1 A).

To validate the sequencing results and explore the correlation with the response to chemotherapy, we examined the levels of miR-378a-3p, miR-378c, miR-378d and miR-4488 in serum-derived exosomes collected from 24 patients who received 4–8 cycles of neoadjuvant chemotherapy regimens containing anthracycline and paclitaxel. Except for miR-181b-5p, which has been confirmed to play a role in chemoresistance and tumorigenesis [14], the results showed that exosomal miR-378a-3p and miR-378d levels were increased in the serum of patients after neoadjuvant chemotherapy compared with before chemotherapy, and this difference was greater in the chemo-insensitive group (Progressive Disease (PD)/Stable Disease (SD)) (miR-378a-3p *p* < 0.01; miR-378d *p* < 0.05) than in the treatment-sensitive group (Complete Response (CR)/Partial Response (PR)) (miR-378a-3p *p* < 0.001; miR-378d *p* < 0.01) (Fig. 1d). These results indicated that the increased serum levels of exosomal miR-378a-3p and miR-378d after chemotherapy were associated with drug resistance (miR-378a-3p *p* < 0.001; miR-378d *p* < 0.01). The RT-qPCR cycle number for miR-378c and miR-4488 was considerably higher than 40, which prevented its analysis. These results suggested exosomal miR-378a-3p and miR-378d as potential biomarkers of chemoresistance in breast cancer patients. More importantly, the five-year survival rate of 854 breast cancer patients from the Cancer Genome Atlas (TCGA) database revealed a poor prognosis among patients with higher miR-378 levels (*p* < 0.05) (Fig. 1e). We also analyzed the relationship between miR-378 expression and stemness indices as an indication of the degree of oncogenic dedifferentiation in 673 breast cancer patients. The tumor epigenetically regulated mRNA expression-based stemness index (mRNAsi) [10] was higher in the miR-378 high expression group (*p* < 0.01) (Fig. 1f).

To further determine the in vivo role of chemotherapy-elicited exosomes in regulating stemness, we conducted gene set variation analysis (GSVA) of the pre- and post-chemotherapy expression data from a previously reported breast cancer cohort in the Gene Expression Omnibus database (GSE28583). We found that post-chemotherapy, tumor mRNAs associated with stemness-related pathways were upregulated compared with the pre-treatment levels (*p* < 0.05) (Fig. 1g). Moreover, we collected tissue samples before and after chemotherapy from 12 patients with poor neoadjuvant chemotherapy efficacy (PD/SD) and found that the expression of β-catenin, NOTCH1, Nanog, SOX2, OCT4 and EZH2 increased after chemotherapy (Fig. 1h). In addition, we found that miR-378 expression was positively correlated with *β-catenin*, *NOTCH1* and *EZH2* expression in breast cancer based on the TCGA database (*n* = 470) (*p* < 0.001) (Fig. 1i).

### Chemotherapy-elicited breast cancer cell-derived exosomes and their cargo RNAs induce chemoresistance in vivo and in vitro

**Fig. 2:**
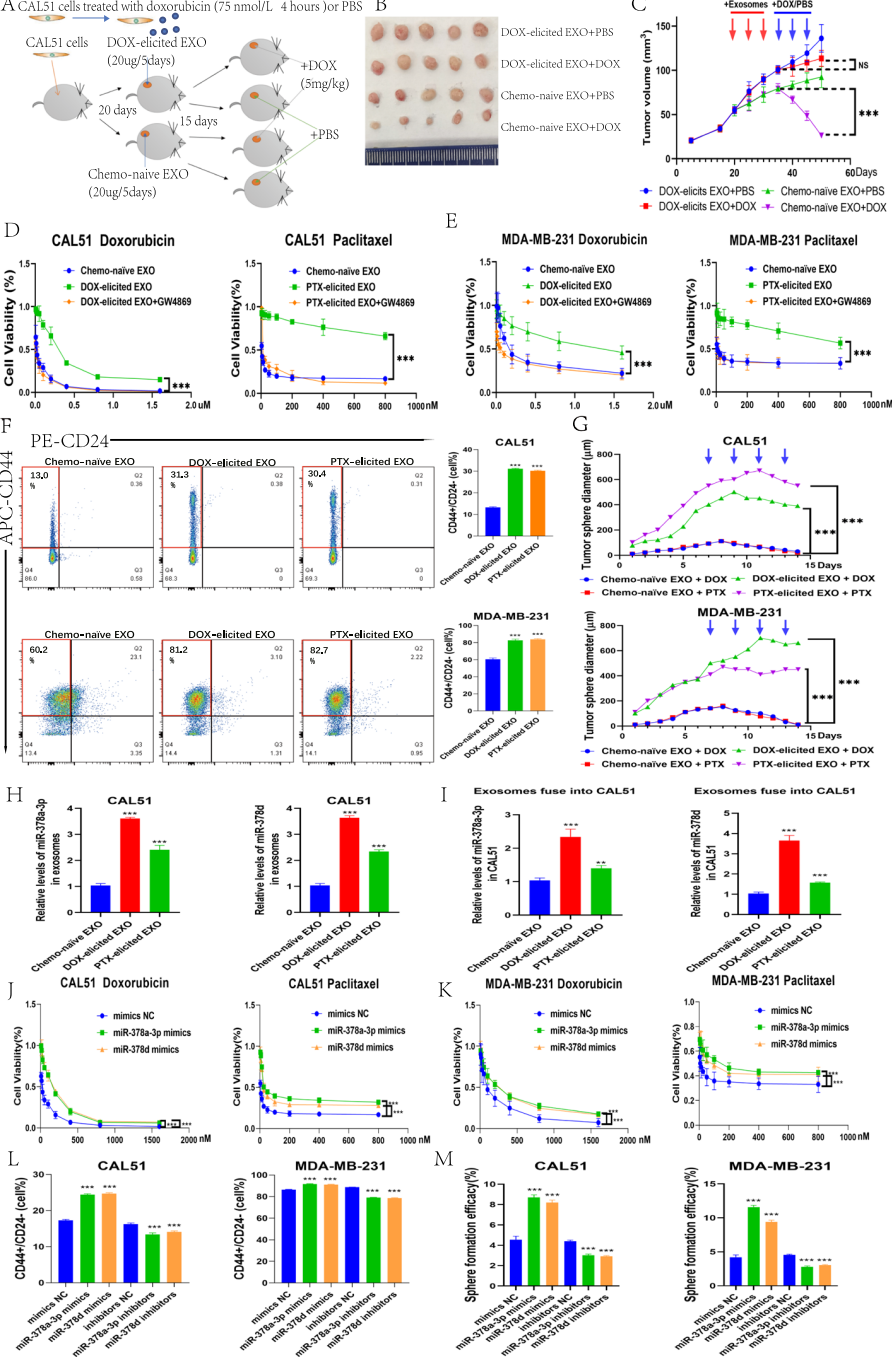
Chemotherapy-elicited breast cancer cell-derived exosomes and their cargo RNAs induce chemoresistance in vivo and in vitro*.* **a** Diagram of animal experiments for chemotherapy-induced drug resistance in exosomes. **b** Images of tumors in mice (*n* = 20). **c** Tumor onset and volume. Red arrows indicate the time of exosome treatments, and blue arrows indicate the time of DOX treatments. **d**, **e** The effects of DOX and PTX on the cell viability of CAL51 and MDA231 cells co-cultured with chemo-naïve exosomes, DOX chemotherapy-elicited exosomes, PTX chemotherapy-elicited exosomes or chemotherapy-elicited exosomes combined with GW4869. **f**, **g** CAL51 and MDA231 cells were cocultured with chemo-naïve exosomes, DOX chemotherapy-elicited exosomes or PTX chemotherapy-elicited exosomes for 48 h and then evaluated using CD44+/CD24- population **f** and 3D sphere formation **g** assays. **h** miR-378a-3p and miR-378d contents in chemo-naïve exosomes and DOX or PTX chemotherapy-elicited exosomes. **i** Changes in miR-378a-3p and miR-378d levels in CAL51 cells co-cultured with chemo-naïve exosomes and DOX or PTX chemotherapy-elicited exosomes for 48 h. **j**, **k** CAL51 and MDA231 cells were transfected with miR-378a-3p or miR-378d mimics or negative control mimics and then exposed to DOX or PTX to evaluate cell viability. **l**, **m** At 48 h after transfection with miR-378a-3p or miR-378d mimics, inhibitors and the corresponding normal control, cells were collected for CD44+/CD24- population **l** and sphere formation **m** assays. *p < 0.05, ** *p* < 0.01, *** *p* < 0.001

We investigated the in vivo effects of chemotherapy-elicited exosomes on the development of chemoresistance in a nude mouse tumor xenograft model. CAL51 xenograft tumors were established in nude mice. Before mice were treated with DOX or PBS, chemo-naïve CAL51 exosomes or DOX chemotherapy-elicited CAL51 exosomes were injected around the tumor (Fig. 2a). Tumors in the groups injected with DOX chemotherapy-elicited CAL51 exosomes continued to grow after treatment with DOX, with minimal differences compared with the PBS-treated group (*p* < 0.05). However, tumors in the chemo-naïve CAL51 exosome-treated groups were significantly smaller after treatment with DOX than in the PBS group (*p* < 0.0001), which indicates that these tumors were unresponsive to chemotherapy at the time of DOX treatment (Fig. 2b, c). These results suggest that DOX chemotherapy-elicited exosomes endow tumors with chemotherapy resistance in vivo.

We next determined the in vitro mechanism by which chemotherapy-elicited exosomes promote the development of drug resistance in BC. We used gradient ultracentrifugation to isolate MCF-7-, CAL51- or MDA-MB-231-elicited exosomes from cell culture media. These exosomes were labeled with PKH26 and co-cultured with corresponding cells. Using an inverted fluorescence microscope, we observed efficient uptake of the extracted exosomes by breast cancer cells (Fig. S2 A).

We observed decreased drug sensitivity to DOX and PTX following chemotherapy-elicited exosome uptake by CAL51 and MDA-MB-231 cells, and this effect was inhibited by the addition of the exosome inhibitor GW4869 [15] (*p* < 0.0001) (Fig. 2d, e). Similar results were obtained with MCF7 cells (*p* < 0.0001) (Fig. S2 B, C). Additionally, the stemness of CAL51 and MDA-MB-231 cells after treatment with different exosomes was assessed by CD44+/CD24- population analysis and sphere-forming assays. We observed a significant increase in the proportion of breast cancer stem cells (*p* < 0.0001) (Fig. 2f, g, Fig. S2 D), and similar results were obtained with MCF7 cells (*p* < 0.0001) (Fig. S2 E, F).

Total miRNA was extracted from an equal amount of exosomes isolated from PBS- or chemotherapy-treated CAL51, MDA-MB-231 or MCF7 cells, and the levels of exosomal miR-378a-3p and miR-378d were then determined by RT-qPCR. We found that both miR-378a-3p and miR-378d were highly expressed in exosomes secreted by breast cancer cells after induction by DOX and PTX chemotherapeutic agents (*p* < 0.001), which suggests that chemotherapy may regulate the exosomal packaging and secretion of these miRNAs (Fig. 2h and Fig. S3 A, B). In addition, when the three types of exosomes were co-cultured with cells for 48 h, RT-qPCR analysis showed that miR-378a-3p and miR-378d expression was higher in cells after co-culture with DOX and PTX chemotherapy-elicited exosomes (*p* < 0.001) (Fig. 2i and Fig. S3 C, D). This suggests that chemotherapy-elicited exosomal miR-378a-3p and miR-378d can be transferred among breast cancer cells.

To investigate the function of exosome-derived miR-378a-3p and miR-378d, CAL51 and MDA-MB-231 cells were transfected with miR-378a-3p and miR-378d mimics or negative control (NC), and post-transfection cells were subjected to drug sensitivity assays, CD44+/CD24- population analyses and sphere formation assays. MiR-378a-3p and miR-378d were found to contribute to the decreased drug sensitivity of cells to DOX and PTX (*p* < 0.0001) (Fig. 2j, k) and increased percentage of cancer stem cells (*p* < 0.0001) (Fig. 2l, m and Fig. S3 E, 4 A). Similar results were obtained with MCF7 cells (*p* < 0.0001) (Fig. S3 E–G, 4 A).

### Chemotherapy-elicited exosomal miR-378a-3p and miR-378d regulated the WNT/β-catenin and Notch stemness pathways by targeting NUMB and DKK3

**Fig. 3:**
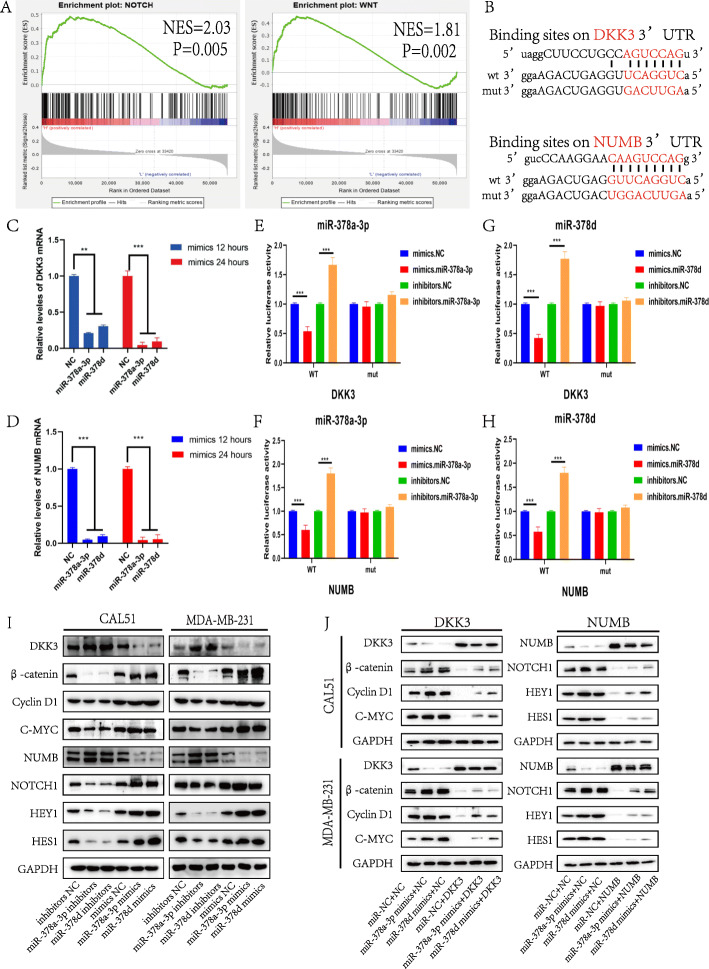
Chemo-elicited exosomal miR-378a-3p and miR-378d regulated the WNT/β-catenin and Notch stemness pathways by targeting NUMB and DKK3. **a** Analysis of miRNA-378 expression data by Gene set enrichment analysis revealed that genes involved in the WNT and NOTCH pathways were significantly enriched. **b** MiRanda prediction of the binding sites of miR-378a-3p/miR-378d within the 3′-UTR of *DKK3/NUMB*. **c**, **d** Relative levels of *DKK3/NUMB* mRNA in CAL51 cells transfected with miR-378a-3p or miR-378d mimics and the corresponding normal control. **e–h** MiR-378a-3p/miR-378d was found to be directly bound to the NUMB/DKK3 3′-UTR using a dual-luciferase reporter assay. **i, j** Western blot analysis of the expression of DKK3, β-catenin, Cyclin D1, C-MYC, NUMB, NOTCH1, HES1 and HEY1 in the different groups. **i** CAL51 and MDA231 cells were transfected with miR-378a-3p or miR-378d mimics, inhibitors and the corresponding normal control for 48 h before western blot analysis. **j** Western blot results showing the expression levels of the indicated proteins in CAL51 and MDA231 cells at 48 h after simultaneous transfection with the DKK3 or NUMB expression plasmid or empty vector and miR-378a-3p or miR-378d mimics or the corresponding normal control. *p < 0.05, ** *p* < 0.01, *** *p* < 0.001

The mechanisms underlying the effects of miR-378a-3p and miR-378d on breast cancer stemness were further investigated. Gene set enrichment analysis (GSEA) was performed on miRNA-378 expression data from TCGA, and the results revealed that the gene expression status was significantly enriched in the WNT (*p* < 0.01) and Notch (*p* < 0.01) pathways (Fig. 3a). The potential target genes of miR-378a-3p and miR-378d were predicted using the miRanda database (*http://www.microrna.org/microrna/home.do*). MiR-378a-3p and miR-378d were predicted to bind to the same site on the same targets. Among these potential targets, DKK3, sFRP1 [16] and SOST [17] are suppressors of the WNT/β-catenin pathway, whereas NUMB [18] is a suppressor of the Notch signaling pathway (Fig. 3b and Fig. S4 B, C). Real-time PCR revealed that the expression of *DKK3* and *NUMB* was inhibited in CAL51 cells transfected with miR-378a-3p and miR-378d mimics compared with the levels detected in the negative control group (*p* < 0.001) (Fig. 3c, d). To demonstrate the direct interaction of miR-378a-3p/miR-378d and DKK3, we established luciferase reporter plasmids containing either the wild-type or mutant 3′-UTR of *DKK3*. Dual-luciferase reporter assays revealed that the inhibitory effect of miR-378a-3p and miR-378d on reporter activity was abolished following transfection with the mutant *DKK3* 3′-UTR plasmid. Similar results were obtained in dual-luciferase reporter assay with miR-378a-3p and miR-378d targeting the *NUMB* 3′-UTR (*p* < 0.0001) (Fig. 3e-h). Western blot analysis demonstrated that the expression of NUMB and DKK3 was strongly inhibited by the overexpression of miR-378a-3p or miR-378d, whereas the expression of stemness-associated proteins, including β-catenin, Cyclin D1, c-MYC, NOTCH1, HEY1 and HES1, was increased. Conversely, miR-378a-3p or miR-378d inhibitors enhanced NUMB and DKK3 expression, and the expression of stemness-associated proteins was decreased (Fig. 3i and Fig. S4 D). Furthermore, after transfection with miR-378 mimics and the DKK3 or NUMB overexpression plasmids, western blot analysis showed that the effect of miR-378a-3p and miR-378d on breast cancer stemness was rescued (Fig. 3j and Fig. S4 E).

**Fig. 4:**
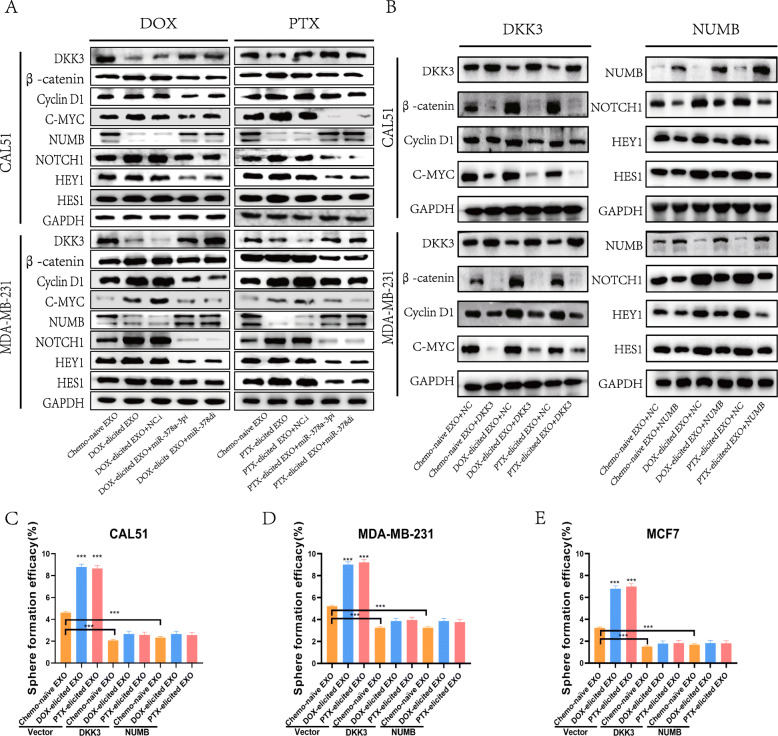
miR-378a-3p/miR-378d inhibitors, the WNT antagonist DKK3 and the Notch antagonist NUMB all inhibit chemotherapy-elicited exosome-induced activation of the stemness pathway. **a, b** CAL51 and MDA231 cells were co-cultured with chemo-naïve exosomes, DOX chemotherapy-elicited exosomes or PTX chemotherapy-elicited exosomes before transfection with miR-378a-3p or miR-378d inhibitors and the corresponding normal control **a** or transfection with the DKK3 or NUMB expression plasmid or empty vector **b** for 48 h; proteins were analyzed by Western blotting. **c–e** MDA231, CAL51 and MCF7 cells were transfected with different plasmids and co-cultured with different exosomes; cells were then collected for sphere formation assays. *p < 0.05, ** *p* < 0.01, *** *p* < 0.001

In addition, after co-culture with chemotherapy-elicited exosomes, western blot analysis revealed that NUMB and DKK3 were strongly suppressed, the WNT/β-catenin and Notch signaling pathways were activated, and the expression of CD44 and ALDH1A1 was increased (Fig. S4 F). Conversely, the activation of WNT/β-catenin and Notch signaling pathways was abolished following transfection with miR-378 inhibitors (Fig. 4a and Fig. S5 A). Furthermore, at 48 h after rescuing DKK3 or NUMB expression, sphere formation assays and western blot analysis showed that the effect of chemotherapy-elicited exosomes on breast cancer stemness was rescued (*p* < 0.0001) (Fig. 4b-e and Fig. S5 B, C). These results demonstrated that chemotherapy-elicited exosomal miR-378a-3p and miR-378d regulate the WNT/β-catenin and Notch stemness pathways by targeting NUMB and DKK3.

### Chemotherapy-elicited exosomes promoted breast cancer chemoresistance by inducing WNT/β-catenin and Notch stemness pathways in vivo

**Fig. 5:**
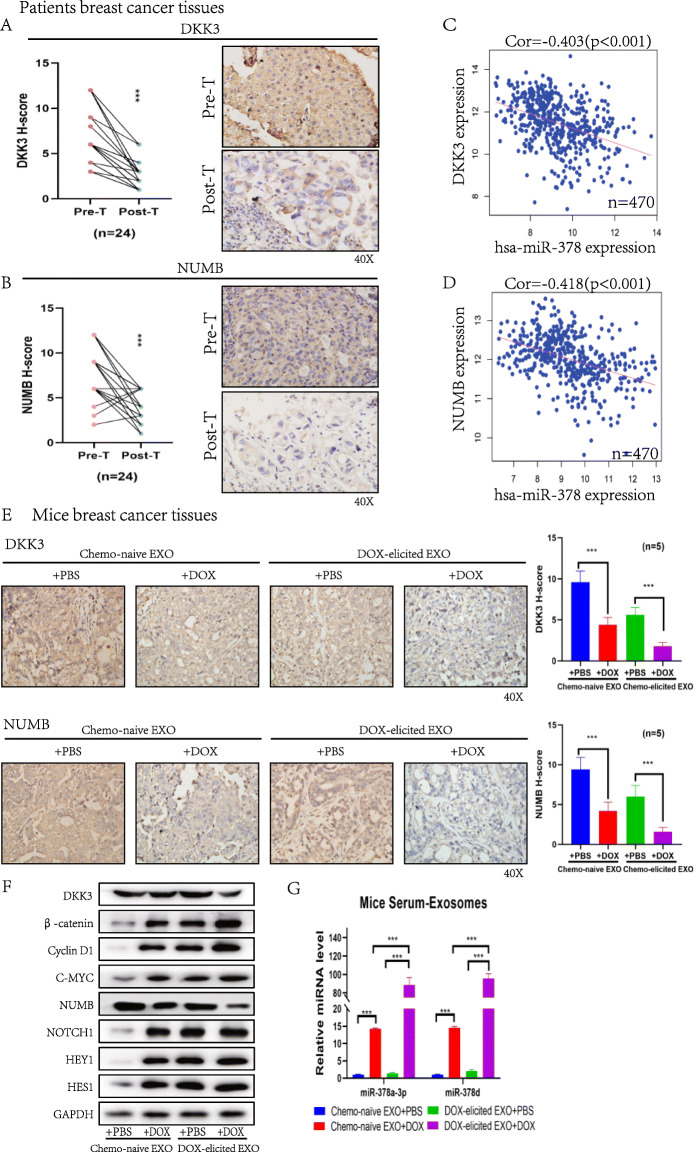
Chemotherapy-elicited exosomes promoted breast cancer chemoresistance by inducing the WNT/β-catenin and Notch stemness pathways in vivo. **a, b** Immunohistochemistry analysis of DKK3 and NUMB in breast cancer patient tissues before and after neoadjuvant chemotherapy in the no response group (*n* = 24). **c, d** The expression level of miR-378 was negatively correlated with *DKK3* or *NUMB* in breast cancer (*n* = 470). **e–g** Mice treated with different exosomes and DOX, as shown in Fig. 2**a**. **e** Immunohistochemistry analysis of DKK3 and NUMB in tumors (*n* = 5). **f** Western blot analysis of the expression of DKK3, β-catenin, Cyclin D1, C-MYC, NUMB, NOTCH1, HES1 and HEY1 in tumors. **g** Serum exosomes were collected and analyzed for miR-378a-3p and miR-378d contents. *p < 0.05, ** *p* < 0.01, *** *p* < 0.001

We collected pre- and post-chemotherapy tissues from patients showing poor neoadjuvant chemotherapy efficacy (PD/SD) to detect changes in DKK3 and NUMB expression using IHC. These data suggest that after chemotherapy, DKK3 and NUMB expression levels are negatively correlated with the response to treatment (*p* < 0.0001) (Fig. 5a, b). In addition, we found that miR-378 expression was negatively correlated with *DKK3* and *NUMB* expression in breast cancer based on the TCGA database (*n* = 470) (*p* < 0.001) (Fig. 5c, d).

We collected tissues from the animals shown in Fig. 2a to detect changes in DKK3 and NUMB expression using IHC, and the results showed that after DOX treatment, DKK3 and NUMB expression levels were suppressed, especially when DOX chemotherapy-elicited exosomes were added (*p* < 0.0001) (Fig. 5e). We also analyzed protein expression, which showed that the stemness-associated proteins β-catenin, NOTCH1, Nanog, SOX2, OCT4 and EZH2 were activated after chemotherapy, and this effect was enhanced by the addition of DOX chemotherapy-elicited exosomes (Fig. 5f). In addition, the levels of miR-378a-3p and miR-378d in exosomes extracted from the serum of mice were higher in the group treated with DOX chemotherapy-elicited exosomes compared with the levels in the groups of chemo-naïve exosomes treated with DOX (*p* < 0.0001) (Fig. 5g).

### Chemotoxicity promotes miR-378a-3p and miR-378d secretion from chemo-treated breast cancer cells by activating the EZH2/STAT3 pathway

**Fig. 6:**
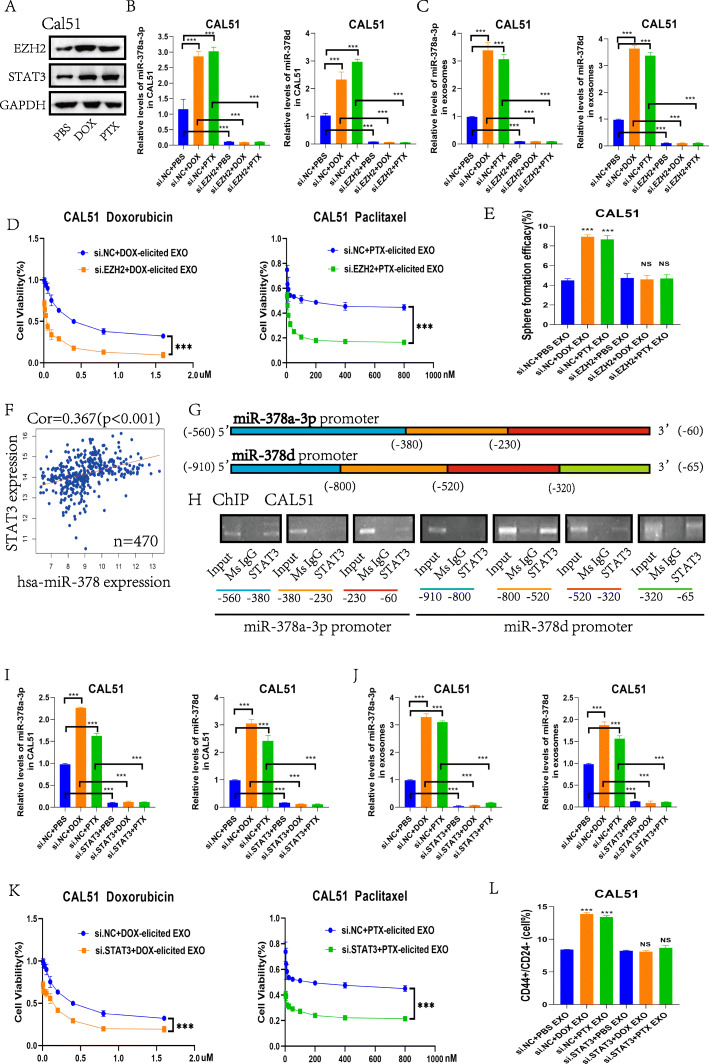
Chemo-toxicity promotes miR-378a-3p and miR-378d secretion from chemo-treated breast cancer cells by activating the EZH2/STAT3 pathway. **a** Western blot detection of changes in the protein expression of EZH2 and STAT3 after 4 h of PBS, DOX or PTX treatment. **b–e** CAL51 cells transfected with si. EZH2 or si.NC for 48 h followed by 4 h of DOX or PTX treatment. **b** Changes in miR-378a-3p and miR-378d expression in CAL51 cells after transfection and drug treatment. **c** Changes in miR-378a-3p and miR-378d expression in exosomes after transfection and drug treatment. **d, e** Exosomes induced by CAL51 cells after transfection and drug treatments were co-cultured with untreated cells. **d** Evaluation of the effect of DOX and PTX on cell viability. **e** Sphere formation assay analysis. **f** The expression level of miR-378 was positively correlated with *STAT3* in breast cancer (*n* = 470). **g** According to the potential binding site, the miR-378a-3p promoter was divided into three segments: − 560 to − 380 for blue, − 380 to − 230 for orange and − 230 to − 60 for red. The miR-378d promoter was divided into four segments: − 910 to − 800 for blue, − 800 to − 520 for orange, − 520 to − 230 for red and − 320 to − 65 for green. (H) Chromatin immunoprecipitation experiments to analyze STAT3 binding to miR-378a-3p and miR-378d promoters. (I–L) CAL51 cells transfected with si. STAT3 or si.NC for 48 h followed by 4 h of DOX or PTX treatment. **i** Changes in miR-378a-3p and miR-378d expression in CAL51 cells after transfection and drug treatment. **j** Changes in miR-378a-3p and miR-378d expression in exosomes after transfection and drug treatment. **k, l** Exosomes induced by CAL51 cells after transfection and drug treatments were co-cultured with untreated cells. **k** Evaluation of the effect of DOX and PTX on cell viability. **l** CD44+/CD24- population analysis. *p < 0.05, ** *p* < 0.01, *** *p* < 0.001

Following the response induced by chemotherapeutic agents in breast cancer cells, both DOX and PTX promoted the expression of EZH2 and STAT3 at 48 h after 4 h of drug treatment (Fig. 6a). Transfection with EZH2 siRNA significantly reduced the levels of miR-378a-3p and miR-378d in CAL51 cells and chemotherapy-elicited exosomes (*p* < 0.0001) (Fig. 6b, c). The induction of drug resistance and stemness by chemotherapy-elicited exosomes was inhibited after transfection with EZH2 siRNAs (*p* < 0.0001) (Fig. 6d, e and Fig. S5 D). Next, we aimed to further validate the role of STAT3 in selectively packaging miR-378a-3p and miR-378d into exosomes and found that miR-378 expression was positively correlated with *STAT3* expression in breast cancer based on the TCGA database (*n* = 470) (*p* < 0.001) (Fig. 6f). The potential transcription factors regulating miR-378a-3p and miR-378d were predicted using the Bioconductor package “TFBSTools” in R language (Fig. S5 E). The miR-378a-3p and miR-378d promoters were divided into fragments according to the predicted binding sites (Fig. 6g). ChIP experiments confirmed that STAT3 binds to the first and third fragments of the miR-378a-3p promoter region and the second to fourth fragments of miR-378d (Fig. 6h). Transfection with STAT3 siRNA also significantly reduced the levels of miR-378a-3p and miR-378d in CAL51 cells and chemotherapy-elicited exosomes (*p* < 0.0001) (Fig. 6i, j). The induction of drug resistance and stemness by chemotherapy-elicited exosomes was also inhibited after transfection with STAT3 siRNAs (*p* < 0.0001) (Fig. 6k, l and Fig. S5 F). These results demonstrate that chemotherapy stimulates activation of the EZH2/STAT3 signaling pathway. Furthermore, STAT3 acts upstream of miR-378a-3p and miR-378d, activating their expression and thus affecting downstream pathways.

The successful construction of EZH2 stable overexpression cell lines was first verified by western blot (Fig. S6. A). A schematic diagram of the experimental design is shown in Fig. S6 B. The EZH2 stable overexpression animal model showed drug resistance after peritumor injection of either chemotherapy-elicited exosomes or chemo-naïve exosomes, and the resistance was more evident in the chemotherapy-elicited exosome injection groups. In contrast, only the chemotherapy-elicited exosome injection groups in the Cal51-MCS animal model showed drug resistance. This experiment shows that both EZH2 overexpression and chemotherapy-elicited exosomes can induce chemotherapy drug resistance (*p* < 0.001) (Fig. S6 C, D). Serum exosomes were extracted from mice, and the miR-378a-3p and miR-378d contents in exosomes were examined by RT-qPCR. The results showed that both EZH2 overexpression and chemotherapy-elicited exosomes contributed to the increase in miR-378a-3p and miR-378d contents in exosomes (*p* < 0.001) (Fig. S6 E).

### Reversal of chemotherapy-elicited exosome-induced chemoresistance in vivo by EZH2 inhibitor tazemetostat

**Fig. 7:**
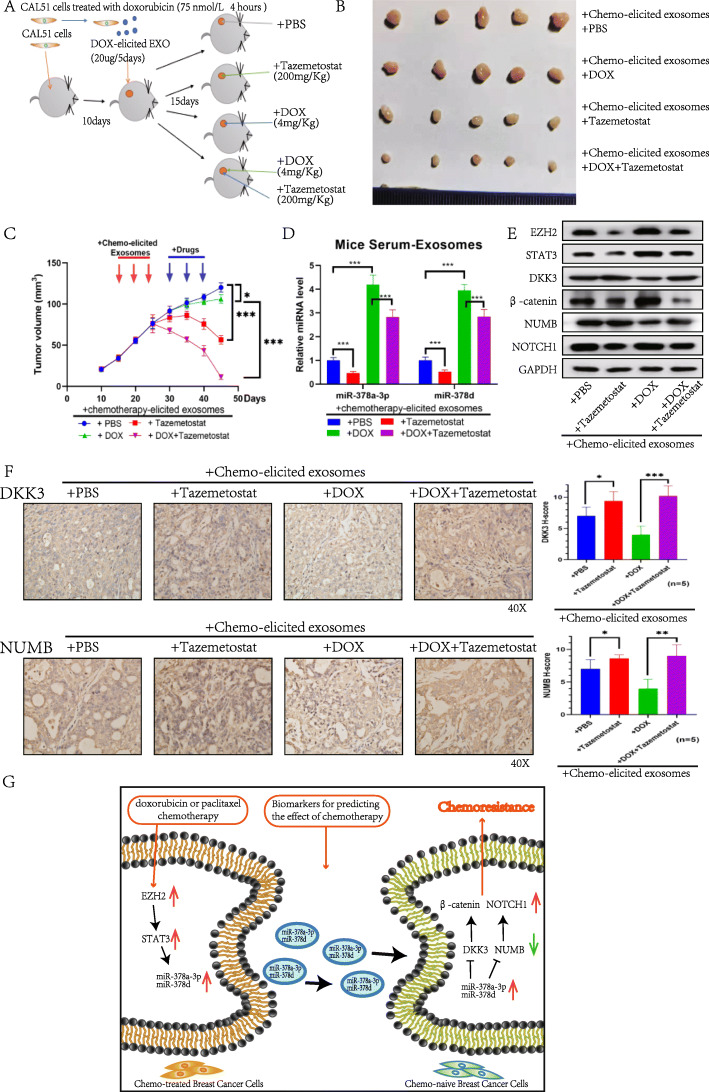
Reversal of chemotherapy-elicited exosome-induced chemoresistance in vivo by EZH2 inhibitor tazemetostat. **a** A schematic diagram of the animal experiments. **b** Images of tumors in mice (*n* = 20). **c** Tumor onset and volume. Arrows indicate the time of treatments. **d** RT-qPCR analysis of miR-378a-3p or miR-378d in serum exosomes in the different treatment groups. **e** Tumor proteins were extracted from the different treatment groups and analyzed by western blotting. **f** Immunohistochemistry analysis of DKK3 and NUMB in the tumors of mice in the different treatment groups (*n* = 5). **g** A proposed model illustrating the role of chemotherapy-elicited exosomal miR-378a-3p and miR-378d in regulating chemoresistance in breast cancer cells. *p < 0.05, ** *p* < 0.01, *** *p* < 0.001

To explore whether blocking this adaptive mechanism by EZH2 inhibitor tazemetostat could reduce the development of chemotherapy resistance and maximize the therapeutic effect, we established a CAL51 tumor xenograft mouse model. DOX chemotherapy-elicited CAL51 exosomes were injected around the tumor, and then mice were treated with PBS, DOX, the EZH2 inhibitor tazemetostat or DOX + tazemetostat. The results showed that DOX chemotherapy-elicited CAL51 exosomes caused drug resistance (*p* < 0.05). In addition, chemoresistance could be reversed by a low dose of tazemetostat alone (*p* < 0.0001), but its effect was enhanced when given in combination with DOX (*p* < 0.0001) (Fig. 7a–c). Tazemetostat treatment significantly reduced miR-378a-3p and miR-378d expression in mouse serum exosomes (*p* < 0.0001). Additionally, the levels of miR-378a-3p and miR-378d in mouse serum exosomes were significantly elevated following DOX treatment (*p* < 0.0001), whereas miR-378a-3p and miR-378d expression were decreased by the addition of tazemetostat (*p* < 0.0001) (Fig. 7d). Western blot analysis showed that Cell culture and transfection DOX decreased NUMB and DKK3 expression but increased β-catenin, NOTCH1, EZH2 and STAT3 expression in CAL51 tumors, and these effects were diminished by the addition of tazemetostat (Fig. 7e). IHC analysis of DKK3 and NUMB expression in the tumors of mice treated with chemotherapy-elicited exosomes and different drugs showed that tazemetostat combined with DOX significantly upregulated DKK3 (*p* < 0.001) and NUMB (*p* < 0.01) expression compared with the DOX treated groups (Fig. 7f). These data collectively suggested that the EZH2 inhibitor tazemetostat could block the stemness-associated signaling induced by chemotherapy-elicited exosomes, thereby contributing to the reversal of therapeutic resistance. Finally, we provide a schematic representation of the biological role of the trans-cellular signaling pathway involving EZH2, STAT3, exo-miR-378a-3p, exo-miR-378d, DKK3 and NUMB in the regulation of breast cancer chemoresistance (Fig. 7g).

## Discussion

In this study, we present novel pathways for the development of drug resistance in breast cancer cells. Chemotherapy activated the EZH2/STAT3 pathway in tumor cells, which caused an increase in miR-378a-3p and miR-378d levels in cells and exosomes. This induced the exosomal delivery of miRNAs targeting DKK3 and NUMB to surviving cells after chemotherapy, and these miRNAs activated the WNT/β-catenin and Notch stem cell-associated pathways, thereby inducing chemoresistance. Anticancer drug resistance remains a major challenge preventing successful cancer treatment. It is increasingly being recognized that exosomes can promote the development of drug resistance through multiple mechanisms. First, they can sequester cytotoxic drugs and reduce the effective concentration at the target site. For example, drug-resistant MCF-7 cell lines overexpress ABCG, and their secreted exosomes also contain ABCG, which promotes drug accumulation in exosomes and drug release [19]. Second, exosomes are an important component of the tumor microenvironment and contribute to the acquisition of therapeutic resistance and tumor progression by mediating communication between cancer cells and stromal cells [20]. Third, exosomes from drug-resistant tumor cells can induce drug resistance in sensitive cells by delivering exosomal mRNAs, miRNAs, long non-coding RNAs and proteins. This phenomenon has been reported in DOX-resistant MCF7 cells [21]. Current research has focused on the role of chemotherapy-elicited exosomes in promoting chemoresistance. For example, it was recently shown that chemotherapy induces the exosome delivery of miR-9-5p, miR-203a-3p and miR-195-5p, which co-target ONECUT2 to promote cancer stemness [6]. In accordance with these findings, our study shows that exosomes elicited in tumor cells after the first cycle of chemotherapeutic stimulation are responsible for the subsequent chemotherapeutic insensitivity mediated by the delivery of miR-378a-3p and miR-378d targeting the WNT pathway inhibitor DKK3 and the Notch pathway suppressor NUMB.

Because exosomal miRNAs are readily detectable and highly stable in several biological fluids, they are potential biomarkers that can be used for the early detection and prognosis of cancer [22]. For example, compared with healthy individuals, exosomal miR-21 is increased in the blood of patients with breast cancer [23] and other types of cancers [24]. In breast cancer, exo-miR-223-3p levels correlate with the pathological stage of lymphatic invasion, pT and pN histological types and early nuclear grade [25]. The analysis of miRNA expression profiles in exosomes isolated from TNBC and HER2-positive breast cancer patients showed that miR-155 and miR-301 were highly predictive of a pathologic complete response [26]. Our results show that exosomal miR-378a-3p and miR-378d levels predict the chemotherapy efficacy in HR-positive/HER2-negative and TNBC patients receiving neoadjuvant chemotherapy containing anthracyclines or paclitaxel.

EZH2 has been shown to influence the development of drug resistance, and EZH2 inhibitors can reverse drug resistance [8] and activate STAT3 [12]. We propose a new pathway by which EZH2 causes chemoresistance. Chemotherapy activates the EZH2/STAT3 pathway, thereby causing increased cytoplasmic and exosome expression of miR-378a-3p and miR-378d. Subsequent uptake of these exosomes by chemotherapy-surviving tumor cells leads to the development of drug resistance. In addition, it has been documented that β-catenin can activate EZH2 expression through synergistic interactions with HMGA2 in triple-negative breast cancer [8]. On the basis of this study, we speculate that exosomal activation of β-catenin induced by chemotherapy can further activate EZH2, thereby enhancing drug resistance, but further validation is needed.

In summary, our findings suggest that chemotherapy-elicited exosomal miR-378a-3p and miR-378d are closely related to the chemotherapy response in a clinical setting. Furthermore, we revealed a novel mechanism of acquired chemoresistance whereby chemotherapy activates the EZH2/STAT3 axis in BC cells, which then secrete chemotherapy-elicited exosomes enriched in miR-378a-3p and miR-378d. These exosomes are absorbed by chemotherapy-surviving BC cells, leading to activation of WNT and NOTCH stem cell pathways via the targeting of DKK3 and NUMB and subsequently resulting in drug resistance. Inhibiting this pathway with an EZH2 inhibitor reverses chemotherapy-elicited exosome-induced drug resistance.

## Supplementary Information

**Additional file 1: Figure S1**. (A) Sequencing analysis of exosomal microRNAs from patient serum exosomes before receiving neoadjuvant chemotherapy, after receiving one cycle of neoadjuvant chemotherapy and after receiving four cycles of neoadjuvant chemotherapy. **Figure S2**. (A) Exosomes were labeled with PKH26 and co-cultured with corresponding cells. (B, C) MCF7 cells were cocultured with different exosomes then exposure to DOX or PTX to evaluated cell viability. (D, E) Images of MDA231, CAL51 and MCF7 cells for 3D sphere formation assay after co-cultured with different exosomes. (F) Images of MCF7 cells for CD44+/CD24- population assay after co-cultured with different exosomes. **Figure S3**. (A-D) Content of miR-378a-3p and miR-378d in chemo-naïve exosomes and DOX or PTX chemotherapy-elicited exosomes and in cells after cocultured with three types of exosomes. (E, F) CAL51, MDA231 and MCF7 cells were cocultured with different exosomes before CD44+/CD24- population assays. (G) MCF7 cells were transfected with miR-378a-3p or miR-378d mimics or negative control mimics then exposure to DOX or PTX to evaluated cell viability. **Figure S4**. (A) MDA231, CAL51 and MCF7 cells were cocultured with different exosomes for sphere formation assays. (B) The binding sites of hsa-miR-378a-3p and hsa-miR-378d are identical. (C) MiR-378a-3p and miR-378d were predicted to bind sFRP1 and SOST. (D) Western Blot analysis of protein expression changes in MCF7 cells after transfection with miRNAs mimics or inhibitors. (E) Western Blot analysis of protein expression changes in MCF7 cells after transfection with miRNAs mimics and DKK3 or NUMB plasmids. (F) Western blot analysis protein expression changes after co-cultured with chemotherapy-elicited exosomes. **Figure S5**. (A) Western Blot analysis of protein expression changes in MCF7 cells after cocultured with chemotherapy-elicited exosomes and transfection with miRNAs inhibitors. (B) MDA231, CAL51 and MCF7 cells were transfected with the DKK3 or NUMB expression plasmids after cocultured with different exosomes, analysis sphere formation assays. (C) Western Blot analysis of protein expression changes in MCF7 cells after cocultured with chemo-elicited exosomes and transfection with DKK3 or NUMB plasmids. (D) CAL51 cells were transfected with si.EZH2 or si.NC then treated with PBS, DOX or PTX and extracted these differently treated exosomes then co-cultured with CAL51cells and analyzed for sphere formation assays. (E) The predicted binding sites of STAT3 binding to miR-378a-3p and miR-378d promoters. (F) CAL51 cells were transfected with si.STAT3 or si.NC then treated with PBS, DOX or PTX and extracted these differently treated exosomes then co-cultured with CAL51cells and analyzed for CD44+/CD24- population assays. **Figure S6**. (A) Western Blot verified successful construction of EZH2 stable overexpression cell lines. (B) A schematic diagram of the experimental. (C) Images of tumor in mice (n = 40). (D) Tumor onset and volume. Arrows indicate the time of treatments. (E) RT-qPCR analysis of miR-378a-3p or miR-378d in serum exosomes in the different treatment groups. **Table S1**. The miRNA mimics, inhibitors and negative control sequences. **Table S2**. Breast cancer patient characteristics. **Table S3**. The primer sequences used in the RT-qPCR. **Table S4**. Western Blot antibodies. **Table S5**. The primer sequences used in the RT-PCR

## Data Availability

The datasets used and analyzed during the current study are available from the corresponding author on reasonable request.

## Abbreviations

BC: Breast cancer
STAT3: Transcription 3
DKK3: Dickkopf 3
EZH2: Zeste homolog 2
DOX: Doxorubicin
PTX: Paclitaxel
pCR: Pathologic complete response
MVBs: Multivesicular bodies
TICs: Stem-like tumor-initiating cells
nSMase: Neutral sphingomyelinase
FBS: Fetal bovine serum
IHC: Immunohistochemistry
CCK-8: Cell Counting Kit-8
OD: Optical density
ChIP: Chromatin immunoprecipitation
WB: Western blot
CR: Complete Response
PR: Partial Response
PD: Progressive Disease
SD: Stable Disease
sr-exosomes: Serum exosomes
TCGA: The Cancer Genome Atlas
mRNAsi: mRNA expression-based stemness index
GSVA: Gene set variation analysis
GSEA: Gene set enrichment analysis
