# Supplementary material for: Chemotherapy-elicited exosomal miR-378a-3p and miR-378d promote breast cancer stemness and chemoresistance via the activation of EZH2/STAT3 signaling
Source: J Exp Clin Cancer Res. 2021 Apr 6;40:120. doi: 10.1186/s13046-021-01901-1 (PMC8022546; doi:10.1186/s13046-021-01901-1)
Supplement: Supplementary file 1 — Additional file 1: Figure S1. (A) Sequencing analysis of exosomal microRNAs from patient serum exosomes before receiving neoadjuvant chemotherapy, after receiving one cycle of neoadjuvant chemotherapy and after receiving four cycles of neoadjuvant chemotherapy. Figure S2. (A) Exosomes were labeled with PKH26 and co-cultured with corresponding cells. (B, C) MCF7 cells were cocultured with different exosomes then exposure to DOX or PTX to evaluated cell viability. (D, E) Images of MDA231, CAL51 and MCF7 cells for 3D sphere formation assay after co-cultured with different exosomes. (F) Images of MCF7 cells for CD44+/CD24- population assay after co-cultured with different exosomes. Figure S3. (A-D) Content of miR-378a-3p and miR-378d in chemo-naïve exosomes and DOX or PTX chemotherapy-elicited exosomes and in cells after cocultured with three types of exosomes. (E, F) CAL51, MDA231 and MCF7 cells were cocultured with different exosomes before CD44+/CD24- population assays. (G) MCF7 cells were transfected with miR-378a-3p or miR-378d mimics or negative control mimics then exposure to DOX or PTX to evaluated cell viability. Figure S4. (A) MDA231, CAL51 and MCF7 cells were cocultured with different exosomes for sphere formation assays. (B) The binding sites of hsa-miR-378a-3p and hsa-miR-378d are identical. (C) MiR-378a-3p and miR-378d were predicted to bind sFRP1 and SOST. (D) Western Blot analysis of protein expression changes in MCF7 cells after transfection with miRNAs mimics or inhibitors. (E) Western Blot analysis of protein expression changes in MCF7 cells after transfection with miRNAs mimics and DKK3 or NUMB plasmids. (F) Western blot analysis protein expression changes after co-cultured with chemotherapy-elicited exosomes. Figure S5. (A) Western Blot analysis of protein expression changes in MCF7 cells after cocultured with chemotherapy-elicited exosomes and transfection with miRNAs inhibitors. (B) MDA231, CAL51 and MCF7 cells were transfected with the DKK3 or NU [file 13046_2021_1901_MOESM1_ESM.docx]

**Supplementary material**

**Supplementary Figures**


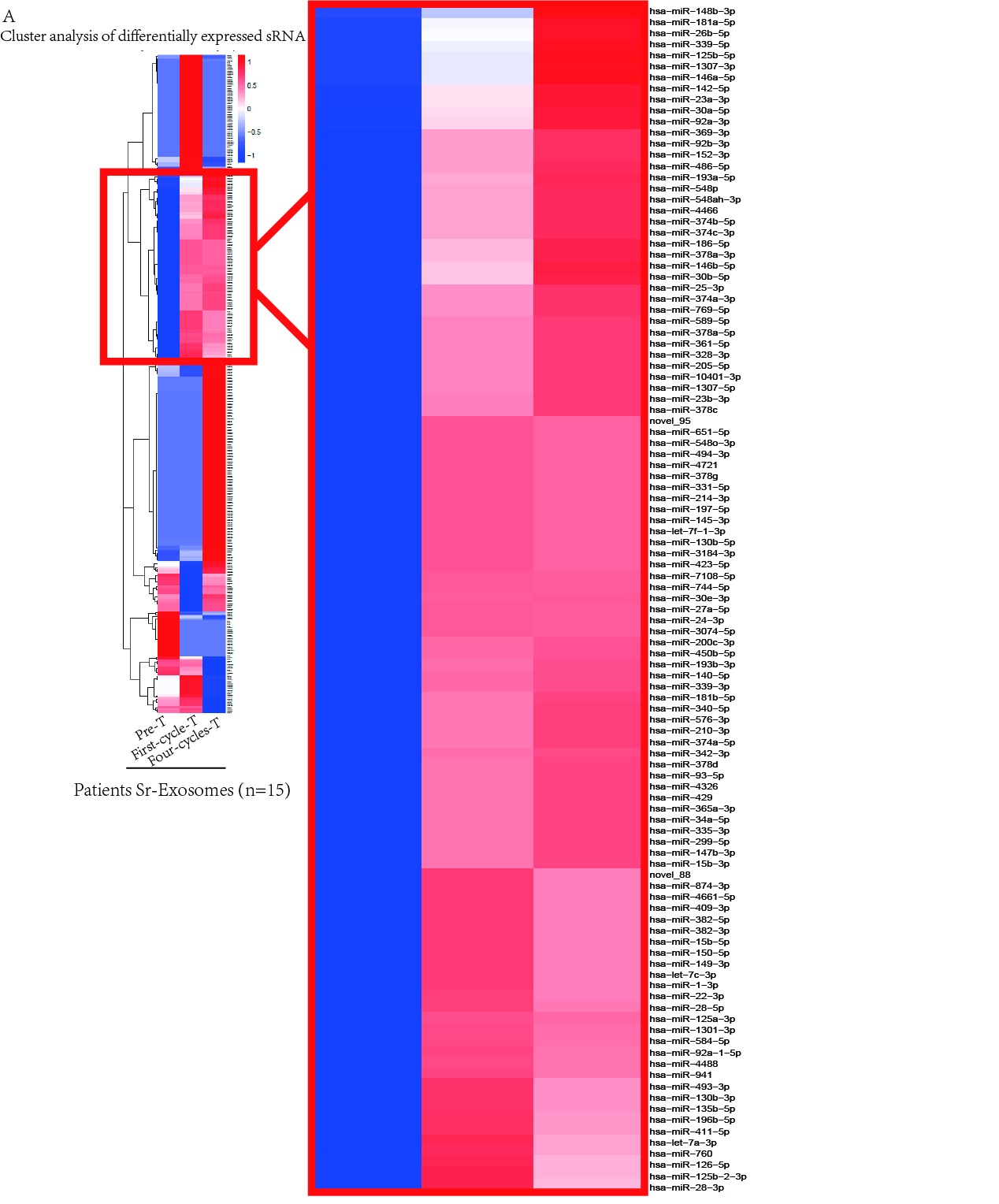


**Figure S1.** （A）Sequencing analysis of exosomal microRNAs from patient serum exosomes before receiving neoadjuvant chemotherapy, after receiving one cycle of neoadjuvant chemotherapy and after receiving four cycles of neoadjuvant chemotherapy.


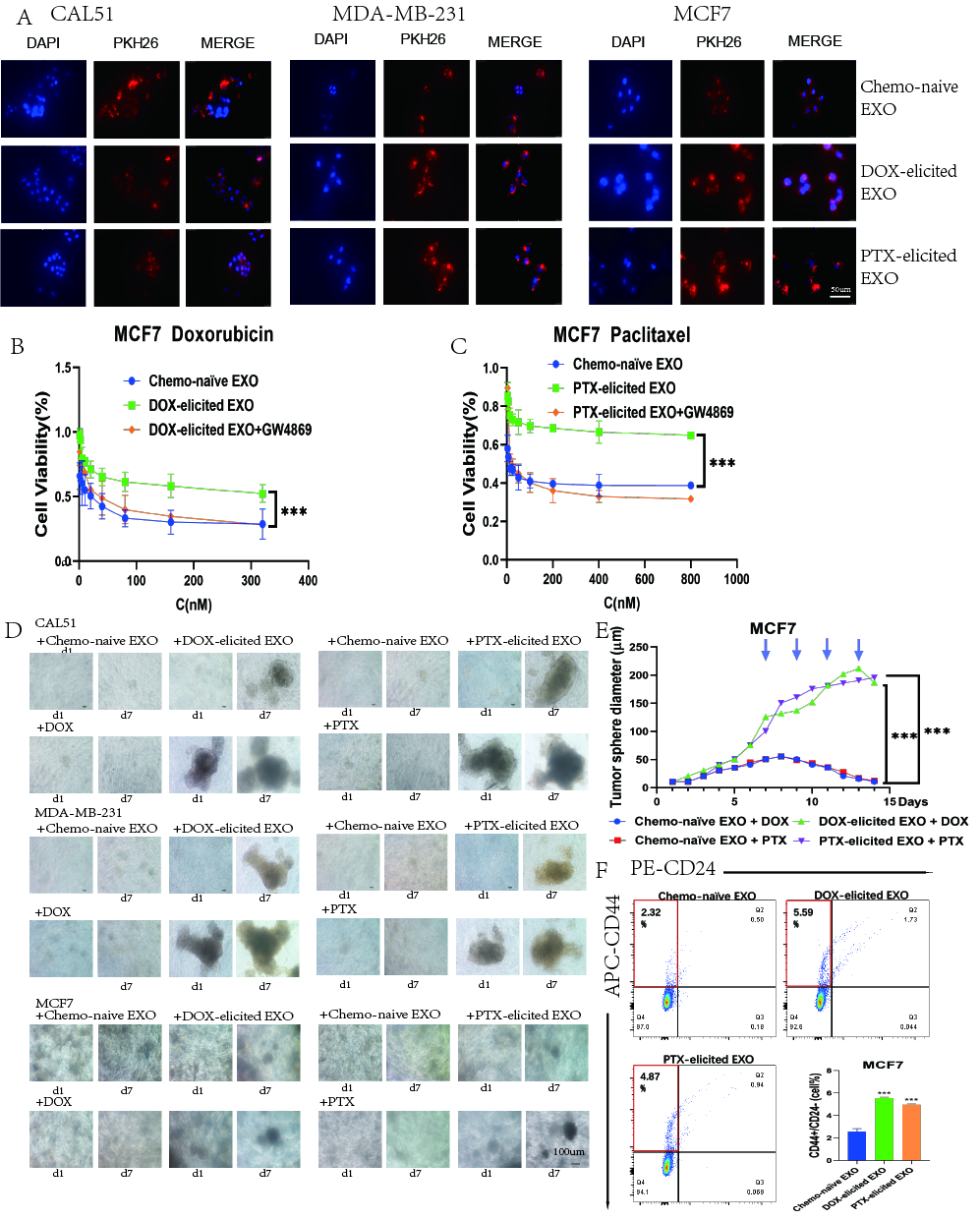


**Figure S2.** (A) Exosomes were labeled with PKH26 and co-cultured with corresponding cells. (B, C) MCF7 cells were cocultured with different exosomes then exposure to DOX or PTX to evaluated cell viability. (D, E) Images of MDA231, CAL51 and MCF7 cells for 3D sphere formation assay after co-cultured with different exosomes. (F) Images of MCF7 cells for CD44+/CD24- population assay after co-cultured with different exosomes.


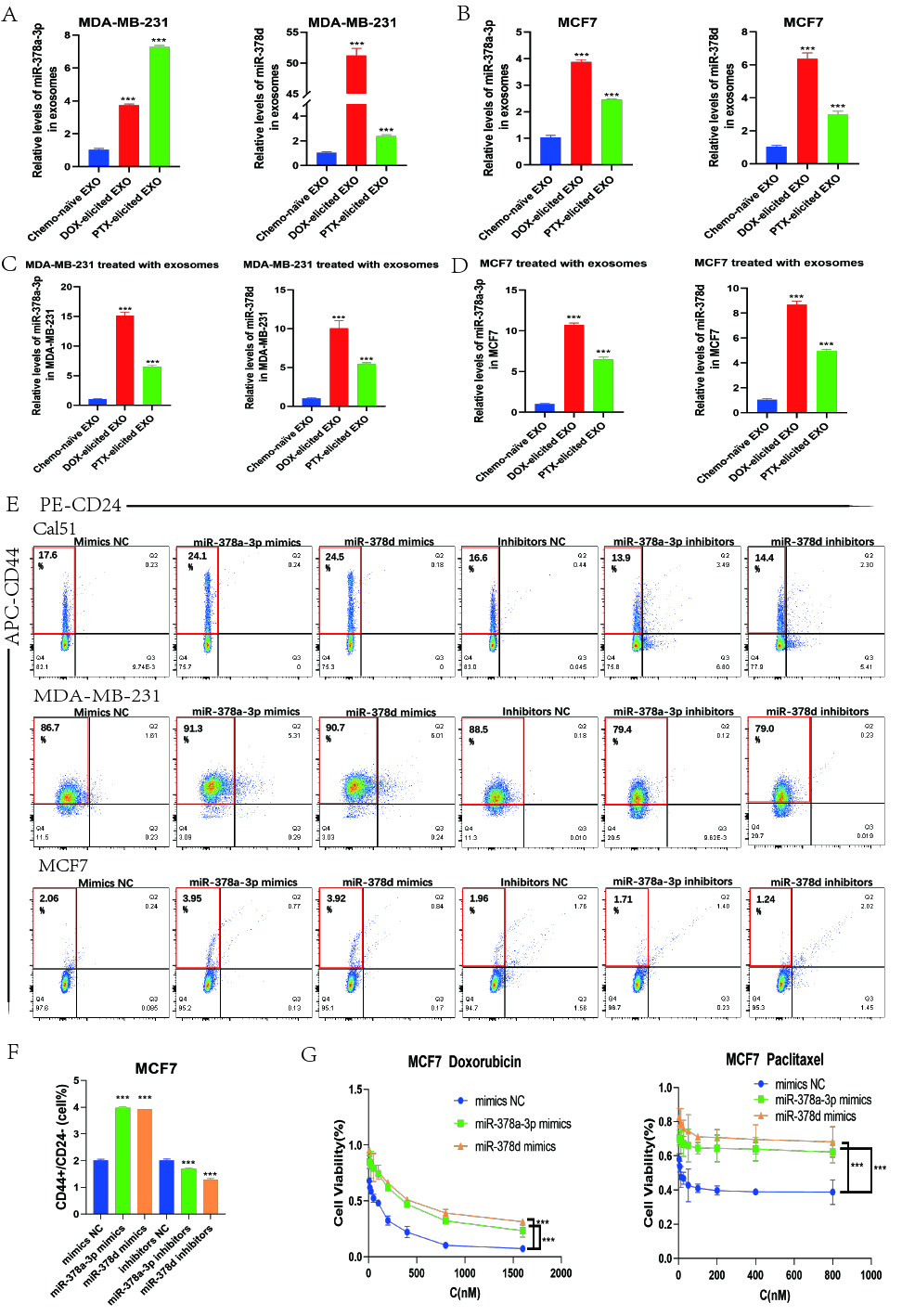


**Figure S3.** (A-D) Content of miR-378a-3p and miR-378d in chemo-naïve exosomes and DOX or PTX chemotherapy-elicited exosomes and in cells after cocultured with three types of exosomes. (E, F) CAL51, MDA231 and MCF7 cells were cocultured with different exosomes before CD44+/CD24- population assays. (G) MCF7 cells were transfected with miR-378a-3p or miR-378d mimics or negative control mimics then exposure to DOX or PTX to evaluated cell viability.


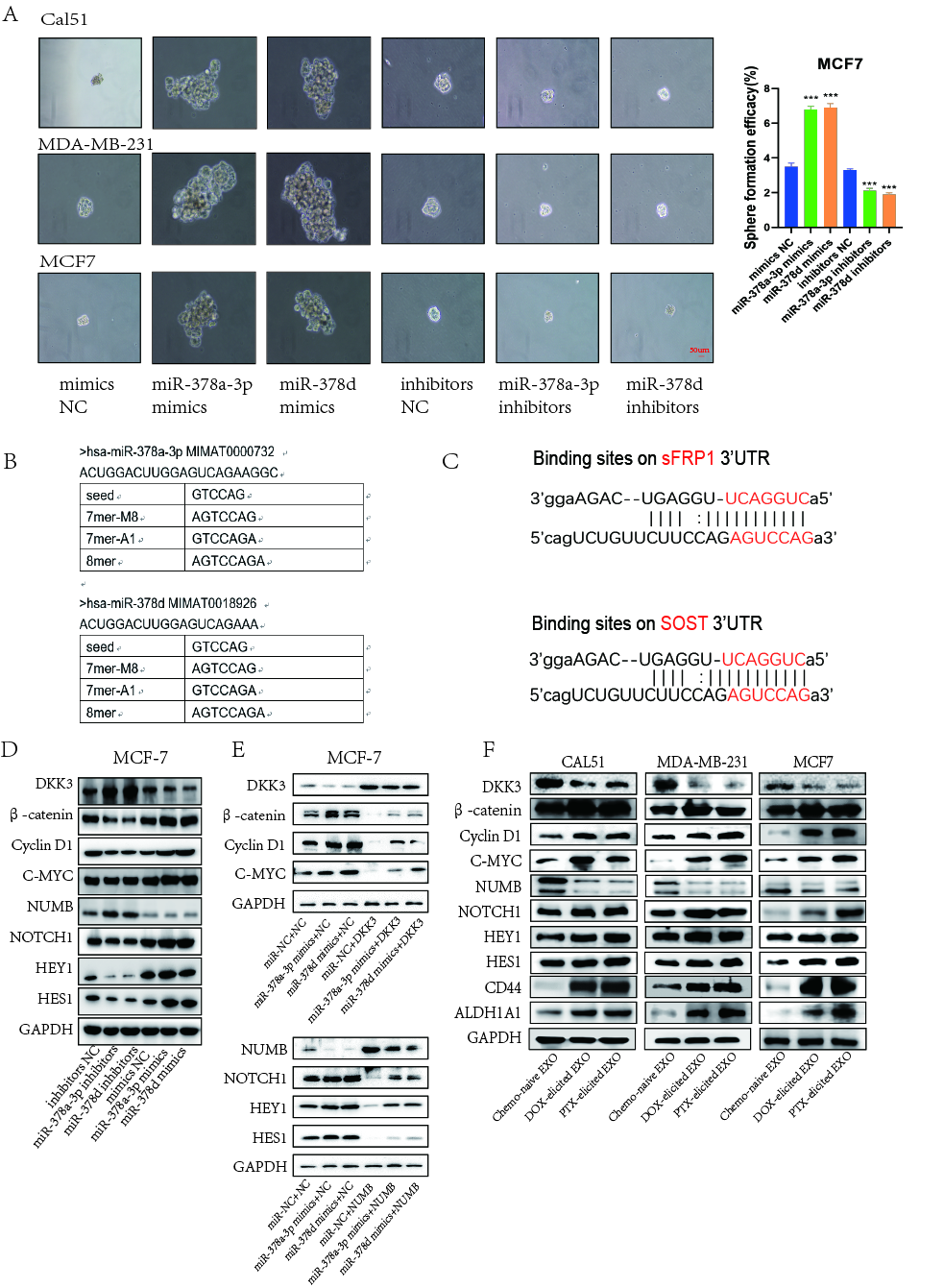


**Figure S4.** (A) MDA231, CAL51 and MCF7 cells were cocultured with different exosomes for sphere formation assays. (B) The binding sites of hsa-miR-378a-3p and hsa-miR-378d are identical. (C) MiR-378a-3p and miR-378d were predicted to bind sFRP1 and SOST. (D) Western Blot analysis of protein expression changes in MCF7 cells after transfection with miRNAs mimics or inhibitors. (E) Western Blot analysis of protein expression changes in MCF7 cells after transfection with miRNAs mimics and DKK3 or NUMB plasmids. (F) Western blot analysis protein expression changes after co-cultured with chemotherapy-elicited exosomes.


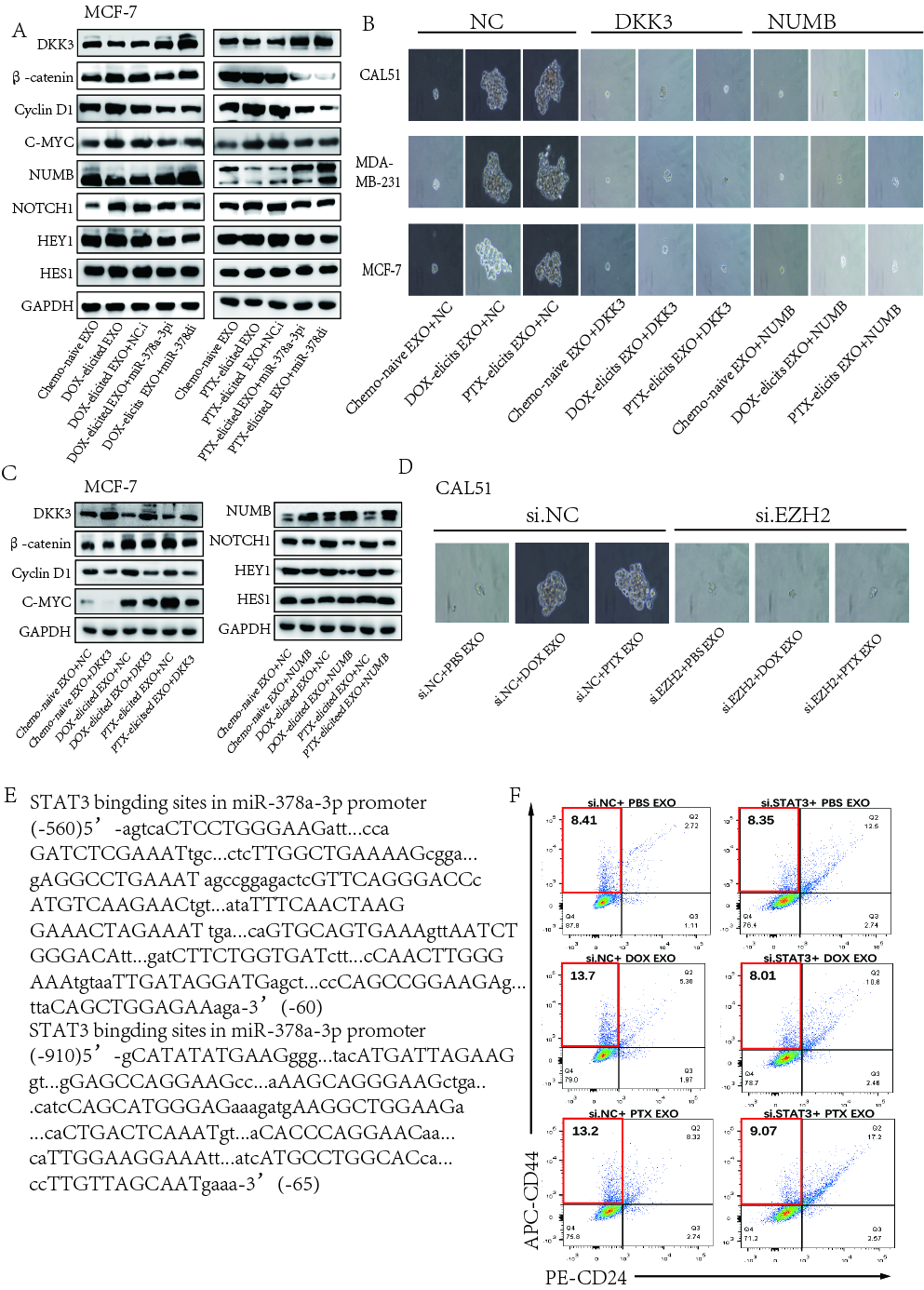


**Figure S5.** (A) Western Blot analysis of protein expression changes in MCF7 cells after cocultured with chemotherapy-elicited exosomes and transfection with miRNAs inhibitors. (B) MDA231, CAL51 and MCF7 cells were transfected with the DKK3 or NUMB expression plasmids after cocultured with different exosomes, analysis sphere formation assays. (C) Western Blot analysis of protein expression changes in MCF7 cells after cocultured with chemo-elicited exosomes and transfection with DKK3 or NUMB plasmids. (D) CAL51 cells were transfected with si.EZH2 or si.NC then treated with PBS, DOX or PTX and extracted these differently treated exosomes then co-cultured with CAL51cells and analyzed for sphere formation assays. (E) The predicted binding sites of STAT3 binding to miR-378a-3p and miR-378d promoters. (F) CAL51 cells were transfected with si.STAT3 or si.NC then treated with PBS, DOX or PTX and extracted these differently treated exosomes then co-cultured with CAL51cells and analyzed for CD44+/CD24- population assays.


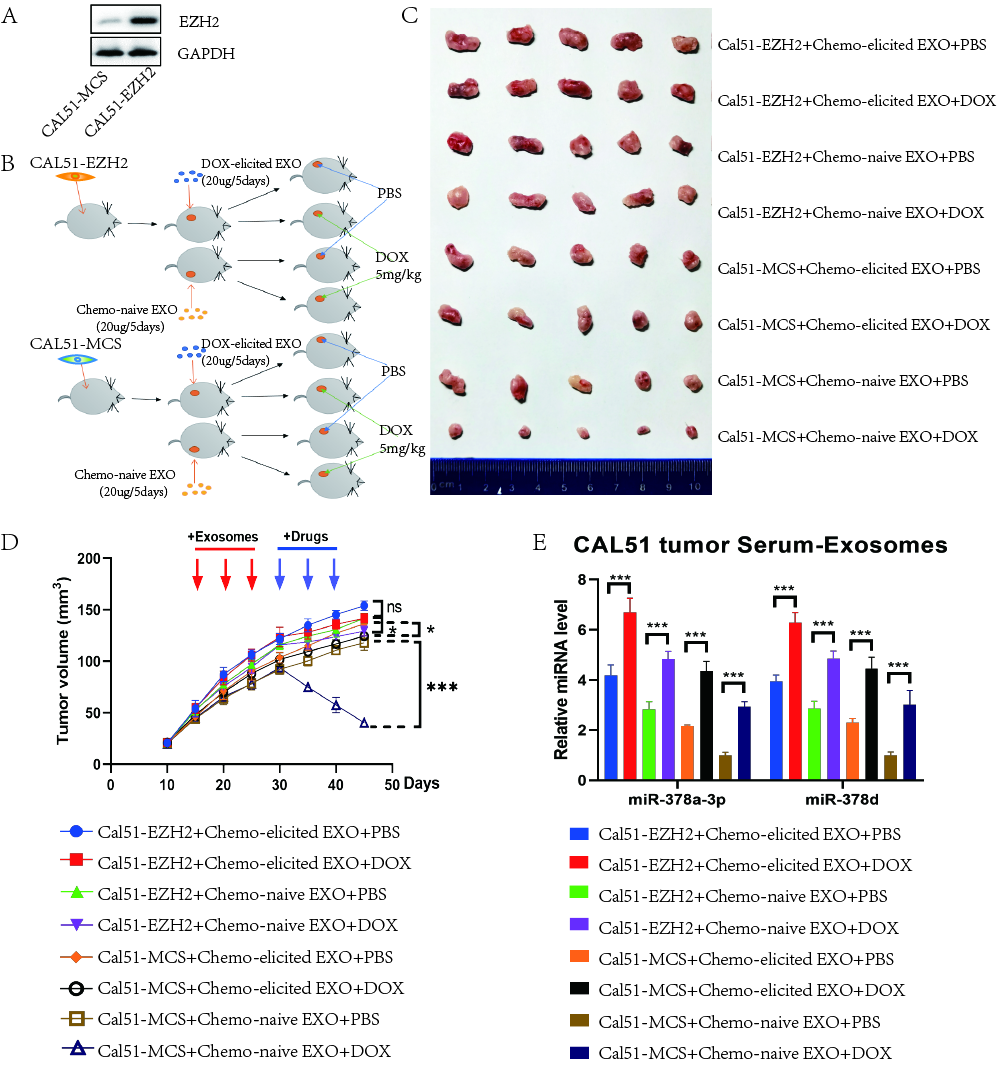


**Figure S6.** (A) Western Blot verified successful construction of EZH2 stable overexpression cell lines. (B) A schematic diagram of the experimental. (C) Images of tumor in mice (n = 40). (D) Tumor onset and volume. Arrows indicate the time of treatments. (E) RT-qPCR analysis of miR-378a-3p or miR-378d in serum exosomes in the different treatment groups.

**Supplementary Tables**

Table S1. The miRNA mimics, inhibitors and negative control sequences.

| Name | Sequences 5’-3’ |
| --- | --- |
| Has-miR-378a-3p mimics | ACUGGACUUGGAGUCAGAAGGCCUUCUGACUCCAAGUCCAGUUU |
| Has-miR-378a-3p inhibitors | GCCUUCUGACUCCAAGUCCAGU |
| Has-miR-378d mimics | ACUGGACUUGGAGUCAGAAAUCUGACUCCAAGUCCAGUUU |
| Has-miR-378d inhibitors | UUUCUGACUCCAAGUCCAGU |
| Mimics negative control | UUCUCCGAACGUGUCACGUTT |
| Inhibitors negative control | CAGUACUUUUGUGUAGUACAA |

Table S2. Breast cancer patient characteristics

| Sample | Subtype  Luminal B is HER2- type | Age | Lymph node metastasis | Tumor size | Chemotherapy regimens | Chemotherapy response  Pathology and clinical | Serum and | Western Blot sample | IHC sample |
| --- | --- | --- | --- | --- | --- | --- | --- | --- | --- |
| 1 | Luminal B | 60 | yes | T4 | TE×4 | Ia / PD | yes | yes | yes |
| 2 | Luminal B | 48 | yes | T1 | TE×4 | Ia-Ib / SD | yes | yes | yes |
| 3 | Luminal A | 62 | yes | T2 | AC×4-T×4 | IIa / PR | yes | no | no |
| 4 | Luminal B | 51 | yes | T3 | TE×4 | Ib-IIa / PR | yes | no | no |
| 5 | Luminal B | 48 | yes | T1 | TE×4 | Ib / SD | yes | yes | yes |
| 6 | Luminal B | 32 | yes | T1 | TAC×6 | 0 / PD | yes | yes | yes |
| 7 | TNBC | 56 | yes | T3 | TE×4-TP×4 | Ia / PD | yes | yes | yes |
| 8 | TNBC | 36 | yes | T1 | TE×4-TP×4 | Ib-IIa / PR | yes | no | no |
| 9 | TNBC | 64 | yes | T2 | TAC×6 | IIb / PR | yes | no | no |
| 10 | TNBC | 57 | yes | T2 | AC×4-T×4 | Ia / PD | yes | yes | yes |
| 11 | TNBC | 63 | yes | T1 | TE×4 | Ia / PD | yes | yes | yes |
| 12 | TNBC | 45 | yes | T2 | TAC×6 | IIb / PR | yes | no | yes |
| 13 | Luminal B | 59 | yes | T4 | TE×4 | Ia / PD | yes | yes | yes |
| 14 | Luminal B | 50 | yes | T1 | TE×4 | Ia / PD | yes | yes | yes |
| 15 | Luminal B | 53 | no | T2 | TAC×6 | IIa / PR | yes | no | no |
| 16 | Luminal B | 48 | yes | T1 | TAC×6 | IIb-IIIa / PR | yes | no | no |
| 17 | Luminal B | 46 | yes | T3 | TAC×6 | IIa-IIb / PR | yes | no | no |
| 18 | Luminal A | 54 | yes | T1 | AT×4 | Ia / PD | yes | yes | yes |
| 19 | Luminal B | 53 | yes | T1 | AT×4 | 0 / PD | yes | yes | yes |
| 20 | TNBC | 43 | yes | T1 | TAC×6 | IIIa-IIIb / CR | yes | no | no |
| 21 | Luminal B | 38 | yes | T2 | TAC×6 | IIa / PR | yes | no | no |
| 22 | Luminal B | 47 | no | T2 | TAC×6 | Ia / PD | yes | yes | yes |
| 23 | Luminal B | 58 | no | T2 | TE×4 | IIa / PR | yes | no | no |
| 24 | Luminal A | 44 | yes | T1 | TAC×6 | Ib-IIa / PR | yes | no | no |
| 25 | TNBC | 45 | yes | T2 | TAC×6 | Ia / PD | no | no | yes |
| 26 | Luminal B | 52 | yes | T2 | TAC×6 | Ib / PD | no | no | yes |
| 27 | Luminal B | 47 | yes | T2 | TAC×6 | Ib / PD | no | no | yes |
| 28 | Luminal B | 53 | no | T1 | TE×4 | Ia / PD | no | no | yes |
| 29 | Luminal A | 62 | yes | T1 | TAC×6 | Ia / PD | no | no | yes |
| 30 | TNBC | 58 | yes | T2 | TE×4 | Ib / PD | no | no | yes |
| 31 | TNBC | 49 | yes | T1 | TE×4 | Ia / PD | no | no | yes |
| 32 | Luminal B | 42 | yes | T3 | TAC×6 | Ia / PD | no | no | yes |
| 33 | Luminal B | 43 | yes | T2 | TAC×6 | Ib / PD | no | no | yes |
| 34 | TNBC | 47 | no | T1 | TAC×6 | Ia / PD | no | no | yes |
| 35 | TNBC | 58 | yes | T2 | AT×4 | Ia / PD | no | no | yes |
| 36 | Luminal A | 64 | yes | T1 | AT×4 | Ia / PD | no | no | yes |

Chemotherapy regimens: T: Taxanes, including paclitaxel, docetaxel, albumin-bound paclitaxel; A: Anthracyclines, including pirarubicin, doxorubicin; E: epirubicin, C: cyclophosphamide; P: Platinum

Pathological assessment of treatment effects: Level 0: Almost no change in cancer cells after treatment; Level I: Ia: Mild changes in cancer cells, and/or visible changes in less than 1/3 of the cancer cells; Ib: Visible changes in 1/3 to 2/3 of cancer cells; Level II: IIa: Significant changes are seen in more than 2/3 of the cancer cells, but significant cancer nests remain; IIb: Very close to grade III efficacy, but a very small amount of cancer cells remain; Level III: IIIa: Complete disappearance of invasive cancer components, but residual intraductal carcinoma; IIIb: Pathological complete response

Clinical chemotherapy response: Complete Response (CR); Partial Response (PR); Progressive Disease (PD); Stable Disease (SD).

Table S3. The primer sequences used in the RT-qPCR.

| Name | primer sequences |
| --- | --- |
| DKK3-forward | ACACAGACACGAAGGTTGGA |
| DKK3-reverse | CGTCTCCCACAGATGTGATA |
| Catenin beta-forward | ATGACTCGAGCTCAGAGGGT |
| Catenin beta-reverse | ATTGCACGTGTGGCAAGTTC |
| C-MYC- forward | TCCTGTCCGTCCAAGCAGAG |
| C-MYC- reverse | CTCAGCCAAGGTTGTGAGGTT |
| Cyclin D1-forward | CGAGGAGCTGCTGCAAATGG |
| Cyclin D1-reverse | CAGAGGGCAACGAAGGTCTG |
| NUMB-forward | AAGGCTTCTTTGGAAAAACTGG |
| NUMB-reverse | CATGGCTCAACCTTTCACCT |
| Notch1-forward | TGAATGGCGGGAAGTGTGAA |
| Notch1-reverse | ATAGTCTGCCACGCCTCTG |
| HES1-forward | TCAGCGAGTGCATGAACGAG |
| HES1-reverse | CATGGCGTTGATCTGGGTCA |
| HEY1-forward | GAGTGCGGACGAGAATGGAA |
| HEY1-reverse | TCGTCGGCGCTTCTCAATTA |
| GAPDH-forward | TGTTCGTCATGGGTGTGAAC |
| GAPDH-reverse | ATGGCATGGACTGTGGTCAT |
| U6-forward | CTCGCTTCGGCAGCACA |
| U6-reverse | AACGCTTCACGAATTTGCGT |
| Has-miR-378a-3p | CTCCTGACTCCAGGTCCTGT |
| Has-miR-378d | ACTGTTTCTGTCCTTGTTCT |
| cel-miR-39-3p | TCACCGGGTGTAAATCAGCTTG |

Table S4. Western Blot antibodies

| Name | Company | product number |
| --- | --- | --- |
| DKK3 | Immunoway | YT1355 |
| Catenin beta | Immunoway | YM3403 |
| C-MYC | Immunoway | YT0991 |
| Cyclin D1 | Immunoway | Yt1172 |
| NUMB | Immunoway | YT5320 |
| Notch1 | Immunoway | YC0067 |
| HES1 | Immunoway | YN2218 |
| HEY1 | Immunoway | YN2962 |
| CD81 | Immunoway | YT5394 |
| Anti-TSG101 | Abcam | Ab83 |
| CD44 | Immunoway | YT6201 |
| ALDH1A1 | Proteintech | 15910-1-AP |
| Nanog | Cell Signaling Technology | 14295-1-AP |
| SOX2 | Cell Signaling Technology | 14962T |
| OCT4 | Cell Signaling Technology | 11263-1-AP |
| GAPDH | Cell Signaling Technology | D16H11 |
| EZH2 | Abcam | ab191250 |
| STAT3 | Cell Signaling Technology | 124H6 |

Table S5. The primer sequences used in the RT-PCR.

| Name | fragments | primer sequences |
| --- | --- | --- |
| Has-miR-378a-3p promoter | -560~-380 forward | AGGCAGGTCCCAGCCTCA |
|  | -560~-380 reverse | CGGGGTACAGTCCAAAGC |
|  | -380~-230 forward | CCCGGATTTTAACTCGA |
|  | -380~-230 reverse | CGGCAAAGATCACCAGA |
|  | -230~-60 forward | AGGATCTTCTGGTGATCTTTGCCGG |
|  | -230~-60 reverse | CCTCGCAGCCTCTTTCT |
| Has-miR-378d promoter | -910~-800 forward | CCATTTGACTCTTGGCTCT |
|  | -910~-800 reverse | CTTTTGAGGTTTTGGGACT |
|  | -800~-780 forward | AACTGAGGAGCCAGGAAG |
|  | -800~-780 reverse | CAGGAGAAGATGGAAGAGC |
|  | -520~-320 forward | ATGGTGCCCACCCAGAT |
|  | -520~-320 reverse | CAGCCCCTCCCACAAAT |
|  | -300~-65 forward | GGCTGGGTTAGGAGTGA |
|  | -300~-65 reverse | TTAGTGCCTGCCTGGAC |
